# Subduction history of the Caribbean from upper-mantle seismic imaging and plate reconstruction

**DOI:** 10.1038/s41467-021-24413-0

**Published:** 2021-07-09

**Authors:** Benedikt Braszus, Saskia Goes, Rob Allen, Andreas Rietbrock, Jenny Collier, Nick Harmon, Tim Henstock, Stephen Hicks, Catherine A. Rychert, Ben Maunder, Jeroen van Hunen, Lidong Bie, Jon Blundy, George Cooper, Richard Davy, J. Michael Kendall, Colin Macpherson, Jamie Wilkinson, Marjorie Wilson

**Affiliations:** 1 Geophysical Institute (GPI), Karlsruhe Institute of Technology, Karlsruhe, Germany; 2 Department of Earth Science and Engineering, Imperial College London, London, UK; 3 National Oceanography Centre, University of Southampton, Southampton, UK; 4 Department of Earth Sciences, Durham University, Durham, UK; 5 School of Earth Sciences, University of Bristol, Bristol, UK; 6 Department of Earth Sciences, University of Oxford, Oxford, UK; 7 Natural History Museum, London, UK; 8 School of Earth and Environment, University of Leeds, Leeds, UK

**Keywords:** Geodynamics, Geophysics, Seismology, Tectonics

## Abstract

The margins of the Caribbean and associated hazards and resources have been shaped by a poorly understood history of subduction. Using new data, we improve teleseismic *P*-wave imaging of the eastern Caribbean upper mantle and compare identified subducted-plate fragments with trench locations predicted from plate reconstruction. This shows that material at 700–1200 km depth below South America derives from 90–115 Myr old westward subduction, initiated prior to Caribbean Large-Igneous-Province volcanism. At shallower depths, an accumulation of subducted material is attributed to Great Arc of the Caribbean subduction as it evolved over the past 70 Ma. We interpret gaps in these subducted-plate anomalies as: a plate window and tear along the subducted Proto-Caribbean ridge; tearing along subducted fracture zones, and subduction of a volatile-rich boundary between Proto-Caribbean and Atlantic domains. Phases of back-arc spreading and arc jumps correlate with changes in age, and hence buoyancy, of the subducting plate.

## Abstract (web summary)

Seismic imaging of subducted plates offers a way to improve plate tectonic reconstructions. Here, Braszus et al. use new ocean-bottom seismometer data from the Lesser Antilles to locate subducted spreading centres and faults thus providing a new understanding of the evolution of the Caribbean plate.

## Introduction

**Fig. 1:**
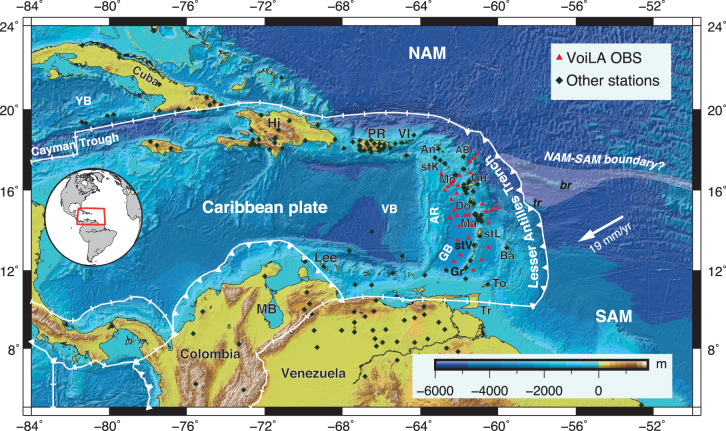
Current tectonic map of the Caribbean. Plate boundaries from Bird^66^. Uncertainty in the position of the NAM–SAM plate boundary is indicated by white shading. Plate motion vector is for Atlantic seafloor relative to the Caribbean plate^26^. Regional seismic networks used for this study include our VoiLA experiment with ocean-bottom seismometers (red triangles) and additional land stations (dark-green diamonds) from which data were added to the global EHB catalogue^53^. An Anguila, AB Antigua and Barbuda (Limestone Caribbees), AR Aves Ridge, Ba Barbados, br Barracuda Ridge, Do Dominica, GB Grenada Basin, Gr Grenada, Gu Guadeloupe, Hi Hispaniola, Lee Leeward Antilles, Ma Martinique, MB Maracaibo Basin, Mo Montserrat, NAM North American plate, PR Puerto Rico, SAM South American plate, stK St Kitts and Nevis, stL St Lucia, stV St. Vincent, To Tobago, Tr Trinidad, tr Tiburon Ridge, VB Venezuela Basin, VI Virgin Islands, YB Yucatán Basin.

Many aspects of the history of the Caribbean plate remain contested because so much of it lies below water and is surrounded by subduction zones (Fig. 1). Caribbean subduction history governs the evolution of the plate’s magmatism and its deformation zones^1–3^. Subduction evolution also created the conditions for the formation of oil-rich basins along the South American coast^4^. The history of subduction along the Caribbean’s western (Central American) margin, of the Farallon plate and its fragments (Cocos, Nazca and Rivera), is relatively well-understood^5–7^, in part because the conjugate to the subducted lithosphere can still be found in the Pacific. By contrast, the northern, eastern and southern margins of the Caribbean have been shaped by subduction along the Great Arc of the Caribbean (GAC) system^8^, and the Proto-Caribbean lithosphere that subducted along this arc system has almost completely disappeared. The geological record preserved along the margins of the Caribbean shows that GAC subduction history involved substantial changes in shape and orientation of the trench, the opening of various back-arc basins, and several generations of volcanic arcs^9–12^, until it evolved into the subduction systems still active today, in particular the Lesser Antilles Arc (LAA). It remains unclear what caused these changes in GAC trench shape and the jumps in arc position. Fragments of the subducted plate have been imaged in the mantle below the Caribbean, but it is debated how these relate to its subduction history^5,13^.

Obtaining better constraints on the evolution of GAC/LAA subduction is also important because it represents one of only two examples of Atlantic lithosphere subduction. Atlantic subduction may differ dynamically from subduction processes around the Pacific Rim on which most of our understanding of subduction zones is based. Whereas Pacific subduction is strongly driven by slab pull^14^, the main driver of convergence along the Lesser Antilles and South Sandwich plate margins appears to be westward motion of the two Americas^15^. Another indication of these different dynamics is that Lesser Antilles subduction represents an endmember in that old plate subducts at an unusually low convergence velocity^16^.

It is commonly accepted that the Caribbean plate originated in the Pacific^8,17^, but relatively little is known about the fate of the Proto-Caribbean lithosphere that previously filled the space between North and South America that the Caribbean plate now occupies. The Proto-Caribbean seaway opened from ~150 Ma as the western arm to the Central Atlantic, as North America moved away from a still joined South America/Africa. Subduction of this lithosphere along an evolving GAC has been suggested to start as early as 135–110 Ma, before the eruption of the Caribbean Large Igneous Province (CLIP), or as late as 85–72 Ma, induced by the CLIP plume^8–10,12,18–20^. This subduction allowed the overriding Caribbean plate to move eastward, relative to the Americas, into its current location. Around 90 Ma, separation of South America and Africa initiated the opening of the Equatorial Atlantic. Spreading at the Proto-Caribbean ridge axis stopped around 70 Ma. Today, only a small sliver of Proto-Caribbean lithosphere remains at the surface (Methods), while along most of the Antillean arc, lithosphere formed at the Mid-Atlantic Ridge is now entering the trench.

Previous *P*-wave travel-time and surface-wave velocity models of the region have imaged a number of distinct slab fragments in the upper mantle below the Greater and Lesser Antilles Arcs and the northern margin of South America^13,21–23^. The latter two studies recognised a gap in the arcuate high-velocity anomaly that underlies the arc from Puerto Rico to Grenada. The gap, in the central Lesser Antilles near Martinique, was attributed to a slab tear along the subducted Equatorial–Atlantic transform boundary between the North and South American plates. However, whilst Van Benthem et al.^13^ imaged it throughout the upper mantle, Harris et al.^23^ imaged the gap only in the upper 200 km. Harris et al.^23^ identified a second shallow slab gap between Hispaniola and Puerto Rico, which was not found by Van Benthem et al.^13^. Imaged patterns in teleseismic shear wave splitting^24^ could be consistent with the existence of both previously proposed tears/gaps, as well as flow around a southern edge of the slab below Grenada.

Even more debated are high-velocity anomalies below the South American margin^13,21–23^. All these studies identified an upper-mantle high-velocity anomaly below the Colombian Maracaibo Basin. Interpretations for this anomaly range from Caribbean lithosphere under-thrust down to 120 km^21^ to a fully developed subducting slab extending down to 660 km^13^.

In the lower mantle, slab material that was subducted eastwards along the Farallon Trench (along the west coast of Central and South America) and material subducted westwards along the GAC Trench have converged, making it even more challenging to unravel slab origins. The first tomography of the region indicated that the GAC slab does not reach below 660 km^25^. However, later work, comparing *P* and *S* tomographic images to a plate reconstruction, found a seismically-fast anomaly, interpreted as the westward-subducted GAC slab, extending down to 1200–1500 km depth, from Hispaniola towards northeast South America^5^. By contrast, the more recent study^13^ found separate lower-mantle high velocities below Hispaniola and northeast South America and attributed them to late Mesozoic subduction below the northern and southern parts of the GAC, respectively.

In this paper, we compare a new *P*-wave travel-time tomography model with improved resolution of the upper mantle (down to 700 km depth) below the eastern Caribbean with a reconstruction of the age structure of Proto-Caribbean lithosphere and predicted subducted slab locations based on the global plate reconstruction by Müller et al.^26^. The combined analysis provides new constraints on the dynamics of the complex past and present tectonics of the Caribbean region.

## Results

### Data, inversion and resolution of tomographic model VoiLA-P19

During our recent VoiLA (Volatile Recycling in the Lesser Antilles) project^27–29^, we deployed a network of broadband ocean-bottom seismometers (OBS) around the Lesser Antilles Arc (Fig. 1). We generate a new *P*-wave travel-time tomographic model, VoiLA-P19^30^, by combining data from this 15-month deployment with an extensive set of recent regional data and an additional 15 years of global data since the last comprehensive model of the region interpreted by Benthem et al. and Amaru^13,31^.

We combine 489 manually picked teleseismic (28–100°) arrivals from our VoiLA experiment with 3732 similarly picked travel times from openly available regional waveform datasets (from the FDSN webservice (https://www.fdsn.org/webservices/, stations in Fig. 1, network codes and references in “Methods”) together with the highest-quality teleseismic picks from the global ISC-EHB travel-time catalogue up to 2016^32^. Using the approach from ref. ^33^, absolute travel times are inverted by simultaneously constraining the 3D velocity structure on a nested global, regional and local grid with increasing spatial resolution (see “Methods” for details). This inversion method helps minimise projecting external structures into our regional domain of interest. The highest resolution (local) grid is divided into 0.75 × 0.75° wide blocks and eight layers between 0 and 660 km depth, ranging in thickness from 40 km at the top to 120 km at the base of the mantle transition zone (Supplementary Fig. 1 and Table 1).

**Fig. 2:**
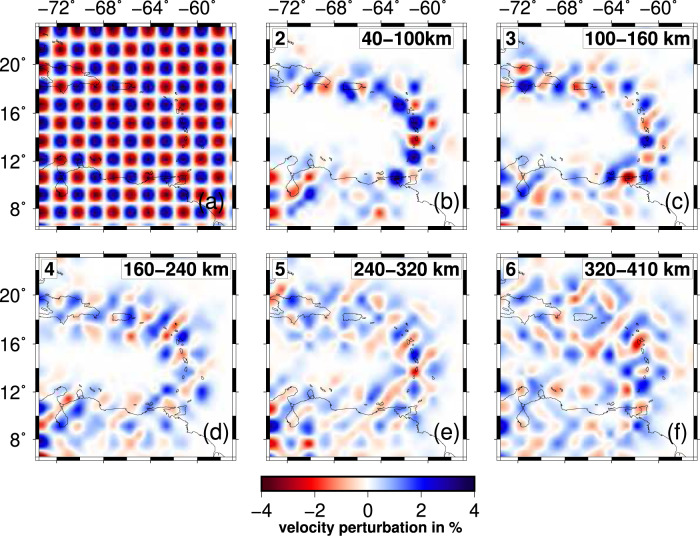
Resolution for model VoiLA-P19, illustrated by checkerboard tests. **a** The input models for the synthetic tests consist of alternating positive and negative velocity perturbations of 10%, either in the odd- or even-numbered layers of the local grid. The horizontal distance between positive and negative anomalies is 1.5°, and their signs are reversed in every perturbed layer. Examples are shown of recovery in (**b**) layer 2 between 40 and 100 km depth, (**d**) layer 4 between 160 and 240 km depth, and (**f**) layer 6 between 320 and 410 km depth for a test where input anomalies are placed only in even-numbered layers. Recoveries in (**c**) layer 3 (100–160 km depth) and (**e**) layer 5 (240–320 km depth) are illustrated for input anomalies in odd-numbered layers. More tests are shown in “Methods” and Supplementary Figs. 5–8.

Coverage provided by the VoiLA dataset complements that of the global catalogue and other regional datasets and results in improved resolution of the upper mantle below the eastern Caribbean (Supplementary Figs. 3, 9). The final spatial resolution is assessed with various resolution tests (Fig. 2 and Supplementary Figs. 5–8, “Methods”). Above 200 km depth, the resolved area covers a wider band around the Lesser Antilles Arc than previous studies, while the centre of the Caribbean Sea remains poorly resolved due to a lack of stations and seismicity. The resolved area expands with depth as an increasing number of horizontal ray paths across the region from earthquakes or to stations along the Caribbean margins. By 300 km depth, the resolved area, recovered with amplitudes of up to ±5%, covers much of the study area. For deeper layers, there is almost homogeneous resolution below the study region, but there is a further reduction in the recovered amplitudes, and the amount of smearing between nodes becomes more significant. Further resolution tests illustrate that synthetic structures, comprising slab anomalies with gaps based on previous models of the region, can be recovered (see “Methods” and Supplementary Figs. 7 and 8). Compared to Van Benthem et al.^13^, resolution is substantially improved in the mantle above 400 km (Supplementary Fig. 9). Compared to Harris et al.^23^, we achieve a more uniform resolution across the region, including in the transition zone.

### Key features of model VoiLA-P19

**Fig. 3:**
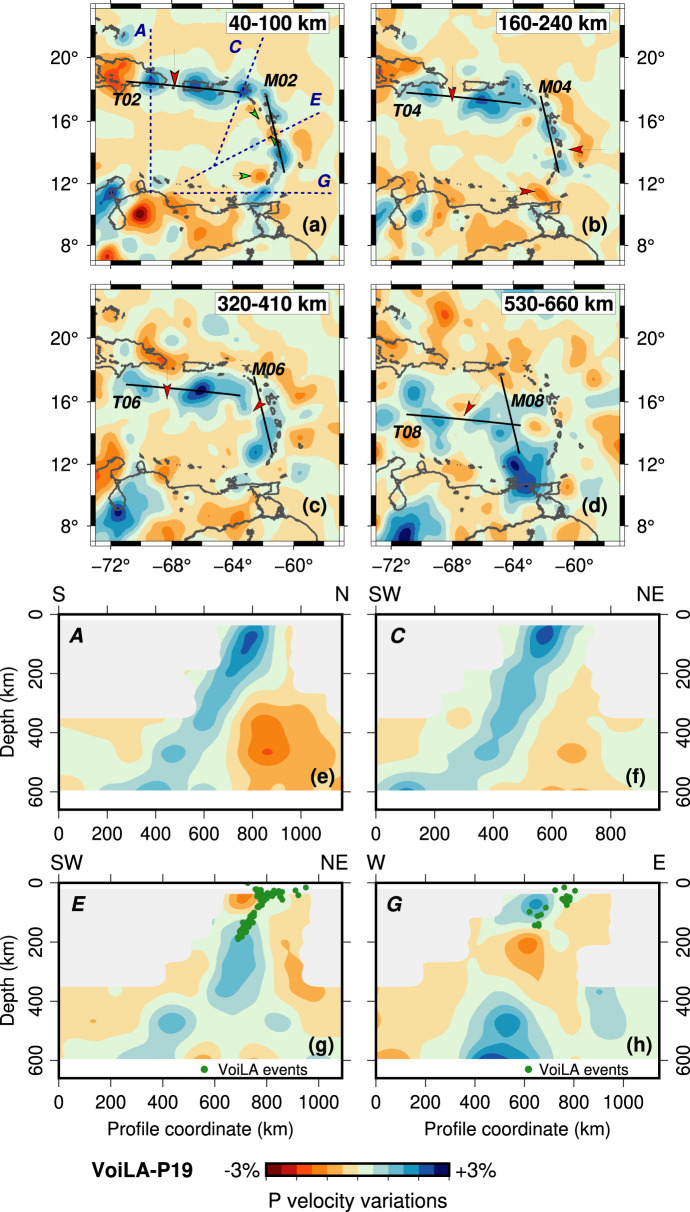
Representative cross-sections through model VoiLA-P19. Velocity anomalies on this and subsequent figures are relative to global model AK135^67^. Plan views are shown in panel (**a**) 40–100 km, (**b**) 160–240 km, (**c**) 320–410 km) and (**d**) 530–660 km depth. Red arrows indicate the possible locations of gaps/reduced anomalies in the slab, green arrows indicate low velocities in the mantle wedge. Dark-blue dotted lines on panel (**a**) show the locations of the cross-sections shown in panels **e**–**h**; solid black lines labelled M02 through M08 and T02 to T08 indicate the location of the dipping cross-sections in Fig. 6. In **e**–**h**, structures are masked (grey) where resolution is limited, i.e. at depths less than 300 km above and behind the slab. Seismicity shown as green dots is from the relocated VoiLA dataset^29^. **e**, **f** Profiles A and C show a continuous slab throughout the upper mantle below Hispaniola and the northern LAA, respectively. **g** The slab below St Lucia follows the seismicity, and above 100–150 km depth is overlain by a particularly slow mantle wedge. At about 400 km depth, there is a ~200-km lateral offset in the slab. **h** Cross-section G south of Grenada contains what looks like a lateral tear in the slab. Slab dip increases significantly below this tear. Further cross-sections in Supplementary Figs. 12, 13.

High-velocity anomalies of model VoiLA-P19 follow the trend of the current Lesser Antilles Arc, LAA (Fig. 3a–d and Supplementary Fig. 12). We find reductions/gaps in these anomalies in similar places as previous studies^13,23^, but depth ranges and relative amplitudes of the anomalies differ, and our comparison with the reconstructed trench positions suggests alternative interpretations. VoiLA-P19 contains the low-velocity anomaly in the slab at around 160–240 km depth below Martinique that Harris et al.^23^ identified (Fig. 3b). In our model, the gap between Hispaniola and Puerto Rico shows up as a reduced high-velocity anomaly that extends deeper, down to at least 400 km (Fig. 3b, c). We also image the gap in the transition zone slab anomaly below the back-arc behind Guadeloupe found by Van Benthem et al.^13^ (Fig. 3c, d). VoiLA-P19 contains particularly low velocities in the mantle wedge above the slab in an approximately linear feature stretching from St Lucia to St Kitts–Nevis–Anguilla and behind St. Vincent (Fig. 3a, g) (consistent with Cooper et al.^34^). A new feature in our model is a break in the slab anomaly between 160 and 320 km depth south of Grenada (Fig. 3b, h). In addition, our new images show more convincingly than previous studies that slab anomalies in the transition zone (400–700 km depth) extend as far west as Hispaniola throughout the transition zone. We also find that there is no connection below the South American margin between the slab anomalies located beneath the Lesser Antilles and the high-velocity anomaly between 250 and 700 km depth below the Maracaibo Basin (around 72°W, 8°N, Fig. 3c, d).

### Plate reconstruction of Proto-Caribbean subduction

To understand the dynamic evolution that led to the complex distribution of high-velocity slab fragments revealed by model VoiLA-P19, we use the most recent global plate reconstruction^26^, with minor modifications to improve the reconstruction of the subducted Proto-Caribbean/Atlantic age structure and to predict slab positions in the mantle.

The Caribbean part of the Müller et al.^26^ reconstruction largely builds on the reconstruction of Boschman et al.^12^ starting 200 Ma, with some modifications^35^. The Müller et al.^26^ model places the Caribbean motions in a global mantle reference frame based on a combination of hotspot tracks, minimisation of trench migration and net lithospheric rotation. An earlier reconstruction^36^ was used in previous interpretations of tomography below the region^13,23^. In the Müller et al.^26^ reconstruction, the opening of the Proto-Caribbean seaway occurred from ~150 Ma through seafloor spreading between the diverging North American (NAM) and South America (SAM)–North–West African (AFR) plates. We will refer to this stage as the Central-Atlantic opening. The break-up between South America and Africa occurred by northward propagation from their southern tips and intersected the Central Atlantic Ridge around 110 Ma, forming a ridge-ridge-transform triple junction that continues to this day. We will refer to this second stage of seafloor spreading as Equatorial–Atlantic opening.

For our regional analysis, two aspects of the global reconstruction were updated (see “Methods”):

1. We refined the position and shape of the Proto-Caribbean mid-ocean ridge axis because of its significance for understanding the evolution of subduction, and for comparison with the mantle seismic velocity anomalies. Symmetrical spreading was assumed without any ridge jumps, such that the ridge axis remains mid-way between the diverging North and South American continents. A minimum number of transform faults were introduced between straight mid-ocean ridge segments to accommodate the break-up geometry in the simplest manner possible (Fz1–3, Fig. 4). Although the number of segments and positions of the transforms are unknown, a few features of Proto-Caribbean seafloor robustly emerge: (i) longer offset transform faults are required in the east than in the west (Fig. 4) to accommodate the break-up geometry around the Bahamas Bank—Demerara Rise; (ii) due to the slow spreading, strong age gradients developed across the Proto-Caribbean Basin, from 0 to 80 Myr over a distance of about 1000 km between the ridge and basin edge.

**Fig. 4:**
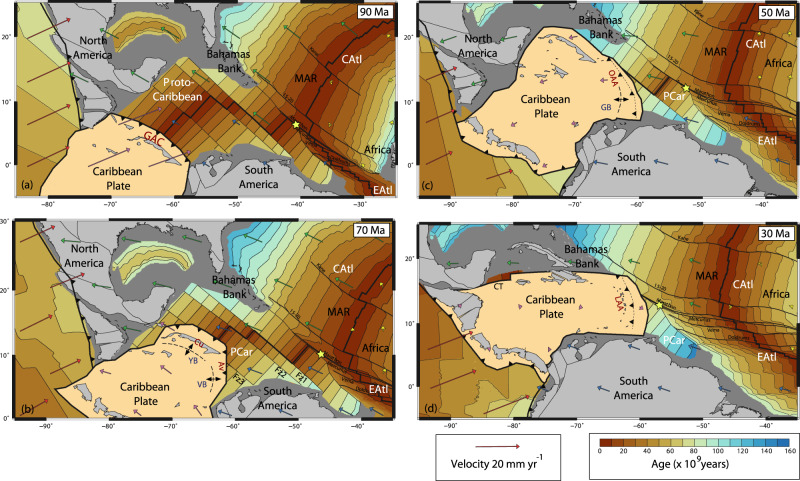
Plate configurations for four time periods. Reconstruction from Müller et al.^26^ with modifications discussed in the text. Velocities are within a mantle reference frame. Oceanic lithosphere ages are at the stated time period (colour scale from ref. ^68^). Continental lithosphere is grey, with light-grey shading showing present-day coastlines for orientation. Key Atlantic fracture zones are labelled. Yellow star indicates where the three oceanic domains (Proto-Caribbean—PCar, Central Atlantic—CAtl, Equatorial Atlantic, EAtl) meet. MAR—Mid-Atlantic Ridge (**a**) 90 Ma—an early phase of subduction along the GAC (Great Arc of the Caribbean) when the Proto-Caribbean spreading ridge was active. **b** 70 Ma—northern and southern parts of the GAC migrate outwards to subduct the oldest Proto-Caribbean lithosphere, accompanied by back-arc spreading in the Yucatán (YB) and Venezualan (VB) Basins behind the Cuban (Cu) and Aves/Leeward Antilles (Av) segments of the arc, respectively. Proto-Caribbean ridge stops spreading; Fz1, Fz2 and Fz3 are hypothetical fracture zones (**c**) 50 Ma—northern (Cuban) segment of GAC inactive after docking against North America, and back-arc spreading initiated in the Grenada Basin (GB), allowing the active arc to migrate east to the Outer Antillean Arc (OAA). **d** 30 Ma—subduction of the large-offset fracture zone(s) around the Bahamas Bank and Demerara Plateau (DP) (marked as Fz1 on panel **b**) led to a rapid younging of the subducting slab, inducing a westward jump of the active arc to the current Lesser Antillean Arc (LAA), while North America-Caribbean motion is accommodated along the newly formed Cayman transform boundary (CT).
2. We modified the geometry of the boundary between the seafloor generated during Proto-Caribbean/Central-Atlantic opening and that generated during Equatorial–Atlantic opening. This improves the prediction of the position of this potentially significant feature on the slab below the Lesser Antilles. We computed synthetic flowlines with combinations of AFR and NAM–SAM rotation poles to match the fracture zone traces observed from modern satellite altimetry data (“Methods”). Our analysis shows that the triple junction that formed when the Equatorial Atlantic and Proto-Caribbean/Central-Atlantic ridges met was originally located at the Mercurius fracture zone. The trace of this feature has been used to separate the two seafloor spreading domains (Fig. 4 and Supplementary Fig. 10, “Methods”).

### Proto-Caribbean plate history and subduction evolution

In the Müller et al.^26^ reconstruction, the onset of westerly-dipping subduction of Proto-Caribbean oceanic lithosphere along the GAC occurred from ~115 Ma. The GAC initiation followed subduction reversal along an intra-American arc and occurred while the active Proto-Caribbean mid-ocean ridge intersected the future trench (Fig. 4a). The subduction of actively spreading seafloor should result in two slab fragments at depth, separated by a slab window. We will refer to these two segments as the northern and southern GAC slabs following ref. ^13^.

As the Proto-Caribbean widened, it comprised increasing amounts of older, and hence more negatively buoyant, oceanic lithosphere (Fig. 4a, b). The GAC lengthened to span the widening Proto-Caribbean, but also by migrating outwards towards the oldest seafloor near the North and South American margins, forming an increasingly curved arc (Fig. 4b, c). Between ~70 and 50–60 Ma, arc migration was facilitated by the formation of two back-arc basins, the Yucatán Basin behind the south-facing subduction below Cuba along the northern GAC e.g.,^10^ and the eastern part of the Venezuelan basin behind the west-facing subduction below the Aves/Leeward Antilles Ridge along the southern GAC^11^. This time also marked the end of divergence between North and South America and hence the cessation of seafloor spreading at the Proto-Caribbean ridge.

Between 60 and 50 Ma, the northern GAC collided with the North American margin and subduction along this part of the GAC ceased. The convergence direction at the continuing Aves Ridge/Leeward Antilles subduction system changed from north-easterly to an easterly orientation (Fig. 4b, c). The Cayman ridge-transform boundary formed to allow eastward movement of the Caribbean plate relative to North America (Fig. 4d). Between ~50 and 35 Ma, the arc jumped eastwards, opening up the Grenada back-arc basin between it and the Aves Ridge. Allen et al.^11^ proposed this was a whole-sale arc jump with the southern portion now buried beneath the Barbados accretionary prism, in contrast to earlier models which only identify the Limestone Caribbees (Fig. 1) as remnants of this system. We refer to this part of the Caribbean arc system as the Outer Antillean Arc (OAA). Finally, at around 25 Ma, the arc jumped back into its own back-arc to form the modern Lesser Antilles Arc (LAA).

An important aspect of the Müller et al.^26^ plate reconstruction is that the northward propagation of the Equatorial Atlantic Ridge was guided by the continental Demerara Rise, such that there is now a sliver of older Proto-Caribbean/Central Atlantic lithosphere between the southern Lesser Antilles and the South American continent^37^ (Fig. 4, “Methods”). The boundary between this and the crust of the Equatorial Atlantic is visible in both satellite gravity and shipboard magnetic data and is clearly identified through the western termination of the fracture zones including and south of Doldrums (“Methods”). This boundary marks a significant (40 Myr) change in oceanic plate age, and hence buoyancy, and may be more significant dynamically than the Equatorial–Atlantic NAM–SAM plate boundary, where the age contrast is negligible.

### Predicting slab locations at depth

To interpret our tomography, we test three endmember cases for slab motions: (1) The slabs sink as if detached from the surface plates, i.e. are laterally stationary within the mantle reference frame. (2) The slabs sink and move as if attached to North and South America (depending on whether subducted north or south of the Proto-Caribbean ridge). (3) A hybrid scenario, where slabs are attached to North or South America until Cuba fully accretes to NAM by 50 Ma, followed by independent sinking. The evolution of slab positions for all cases is shown in Supplementary Fig. 11. In cases (2) and (3), most of the predicted slab positions are located well west of any eastern Caribbean slab anomalies and many positions are located west of the anomalies that are commonly attributed to the Farallon subduction, which occurred west of GAC subduction (Supplementary Figs. 11 and 16). We, therefore, focus our subsequent comparison on case (1) which yields slab predictions in closest proximity to the observed locations of positive seismic velocity anomalies in the Caribbean and South American mantle (Supplementary Figs. 11–15 and Movie).

It may seem surprising that the tomography is best fit by the case (1) detached slab-motion model. However, dynamic models show that relatively low slab strength is required to satisfy observations of slab shapes and plate motions e.g.^38,39^, which would hamper significant lateral slab pushing as required in cases 2 and 3, e.g., ref. ^40^. Vertical sinking is also consistent with the, on average, steep upper-mantle dip of present-day slabs (70–80°), in particular in ocean–ocean subduction zones^16,41^. Various other comparisons of tomography and plate reconstructions also find good fits when vertical sinking within the mantle reference frame is assumed^5,7,42^. Finally, we now find several tears in the GAC slab that likely reduce the connectivity between the subducted slab and the North and South American plates.

**Fig. 5:**
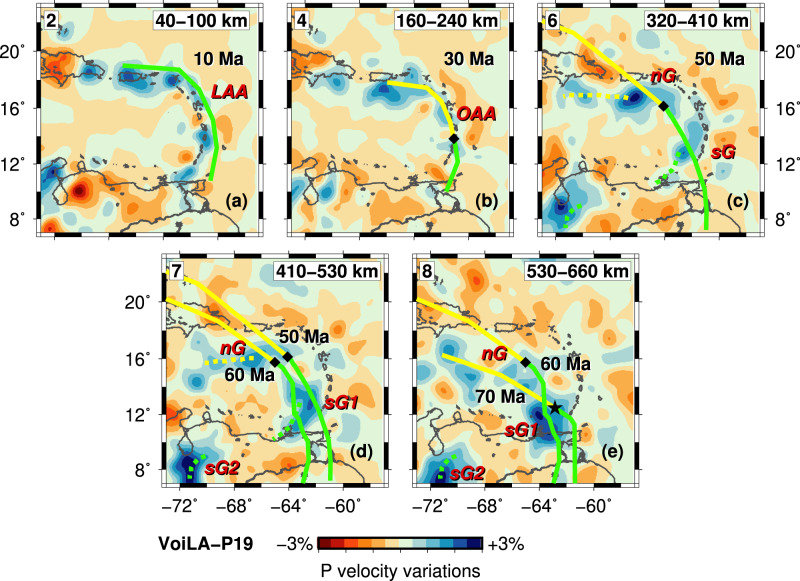
Plan views of *P*-wave velocity anomalies of model VoiLA-P19 with reconstructed slab positions. **a** Slice at 40–100 km depth, with the position of the slab subducted at 10 Ma along the current Lesser Antilles Arc (LAA), **b** Slice at 160–240 km with slab subducted along the Outer Lesser Antilles Arc (OAA) at 30 Ma. **c** Slice at 320–410 km with the position of slab subducted along the GAC/OAA at 50 Ma. **d** Slice at 410–530 km with positions of slab subducted along the GAC 50 and 60 Ma. **e** Slice at 530–660 km with positions of slab subducted 60 and 70 Ma. In yellow: Cuban part of the GAC. In green: Aves/Leeward Antilles part of the GAC. Slab positions are dashed to indicate possible deformation post subduction. The black diamond shows the location of the extinct spreading centre which is a likely location of a tear between the two slabs, black star indicates where the spreading centre subducted while still active, forming a slab window. Red labels mark our interpretation of the high-velocity anomalies as derived from subduction along the LAA, OAA or northern (nG) and southern (sG1 and sG2) part of the GAC.

**Fig. 6:**
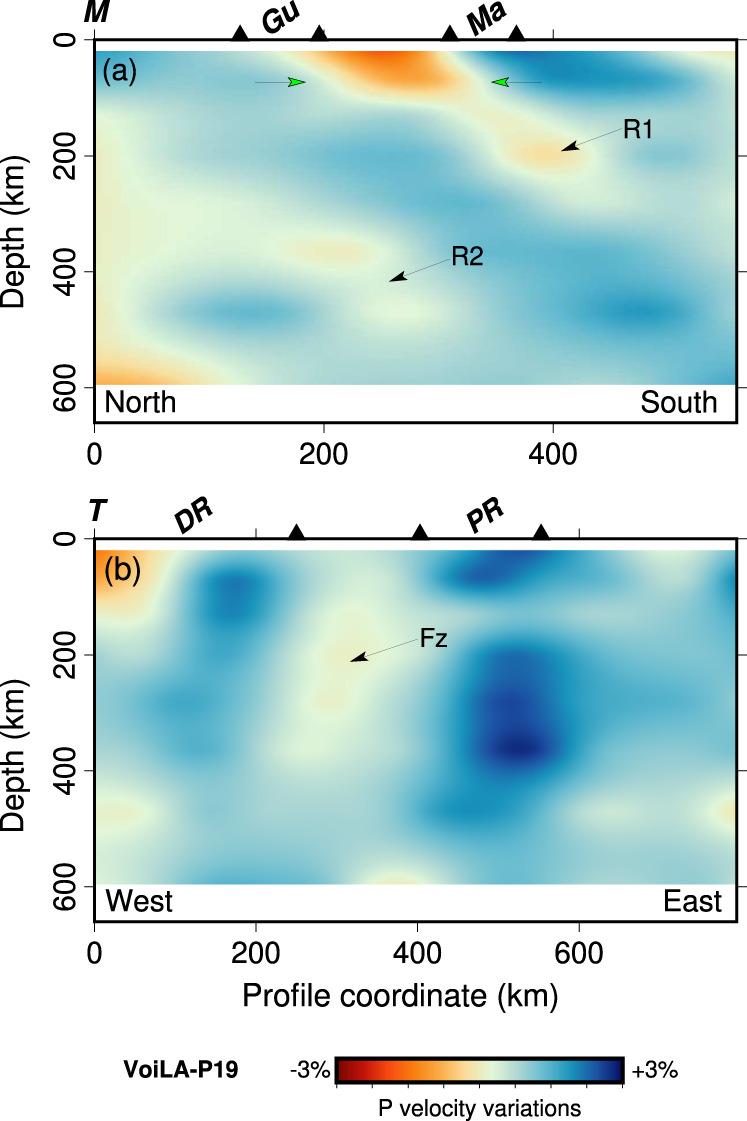
Two dipping cross-sections along the upper-mantle slab anomalies. Locations of the cross-sections are indicated on Fig. 3a. **a** Cross section M along the Lesser Antilles Arc. Gu—Guadeloupe, Ma—Martinique. **b** Cross section T along the slab from Hispaniola to St Kitts. DR—Dominican Republic (Hispaniola), PR—Puerto Rico. Proposed interpretations are marked with arrows: green arrows indicating hydrated mantle wedge, R1, R2 positions of the subducted Proto-Caribbean ridge and Fz—location of a potential tear along a fracture zone.

**Fig. 7:**
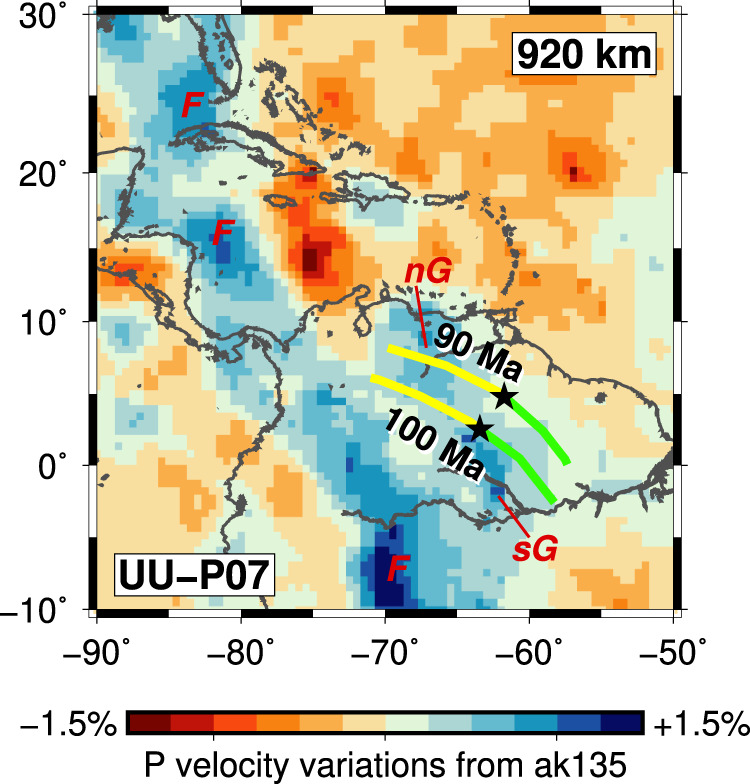
Shallow lower-mantle structure with reconstructed trench positions. Slice at 920 km depth through tomographic model UU-P07^13, 31^. Red labels mark our interpretation of the high-velocity anomalies as derived from the subduction of: F Farallon, nG and sG northern and southern parts of the GAC. Coloured lines show our reconstructed positions of the trench at 90 and 100 Ma. The yellow lines (nG) and green lines (sG) indicate subduction to the northwest and southeast of the actively spreading Proto-Caribbean ridge (black stars), respectively.

Comparison of the reconstructed slab positions with the high-velocity features in VoiLA-P19 shows that the best correspondence is achieved for a sinking velocity of 0.8–1 cm/yr (Figs. 5, 6, 7 and Supplementary Figs. 12–15), i.e., a seismic anomaly interpreted to be a slab at around 320 km depth would have been subducted ~40 Ma ago. However, while the sinking rate is similar to global estimates for lower-mantle sinking^43^, the rate is rather low for upper-mantle sinking^44^, and may be the result of a significant amount of vertical buckling, possibly in response to along-strike slab bending in the mantle transition zone.

## Discussion

### Subduction along the Lesser Antillean Arcs (~50–0 Ma, 400–0 km depth)

**Fig. 8:**
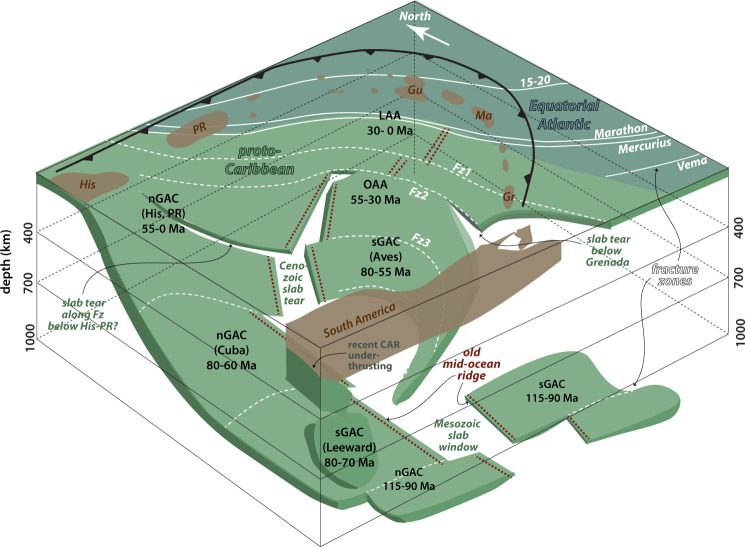
3D sketch of Proto-Caribbean slab fragments in the mantle below the eastern Caribbean as inferred from this study. For reference, some of the larger islands are marked on top (His—Hispaniola, PR—Puerto Rico, Gu—Guadeloupe, Ma—Martinique, Gr—Grenada), an approximate South American coastline is drawn and an approximate north is indicated. Lithosphere produced in the Equatorial Atlantic (teal coloured, with fracture zones in solid white lines) only recently entered the trench below the Lesser Antilles Arc (LAA). Most of the subducted lithosphere in the mantle below the islands was produced during Proto-Caribbean spreading (pale green, with dashed white lines for hypothetical fracture zones). There are gaps in the slab structure where the Proto-Caribbean mid-ocean ridge (marked with bold-red dashed lines) subducted while it was still spreading, leading to a slab window during subduction at the Northern and Southern Great Arc—nGAC and sGAC—before 70 Ma, or it subducted before the lithosphere had much time to cool, leading to further tearing until 40–50 Ma. There is a lateral tear at ~200 km depth in the slab below Grenada, which we propose may follow one of the hypothesised fracture zones, along which tearing occurred during subduction along the Outer Antilles Arc (OAA). In addition, the slab is probably contorted by the northward push of South America leading to further tearing along a fracture zone below Hispaniola/Puerto Rico, and one in the transition zone behind the central arc and folding in the transition zone as slow subduction entered increasing amounts of material at approximately the same location in the mantle. Other parts of the slab that subducted below the sGAC may have sheared off along the cratonic South American margin and could currently be residing in the upper mantle below the coast of Venezuela.

The 30–0 Ma phase of LAA subduction shows up as a continuous anomaly in VoiLA-P19 between the surface and about 250 km depth, stretching from Grenada to the eastern half of Hispaniola, coincident with the Benioff seismicity (Fig. 5a, b and 3 and Supplementary Figs. 12, 13). Based on comparison with the reconstructed slab positions, we propose interpretations for the three gaps within the slab anomalies. Low velocities in the slab below Martinique coincide with where the extinct Proto-Caribbean ridge subducted 30–40 Ma (Figs. 5b, 6, 8 and Supplementary Fig. 12). Given the distribution of slab seismicity (which extends down to 180 km depth) does not indicate the existence of a slab tear along this anomaly^29^, the gap probably reflects a thin plate and possibly excess hydration. After the docking of Cuba, around 50 Ma, the last remaining segment of Proto-Caribbean lithosphere north of the extinct ridge subducted very obliquely below Hispaniola and Puerto Rico producing the current shallow slab below these islands. Comparison with the reconstruction (Fig. 4 and Supplementary Movie) indicates that the reduction in slab anomaly between Hispaniola and Puerto Rico in the shallow mantle may be a tear or low-velocity material along one of the proposed fracture zones in the Proto-Caribbean (Fig. 8). The gap in the slab anomaly at about 200-km depth south of Grenada (Fig. 3h) lies below the maximum depth of Benioff seismicity. We interpret this gap as a lateral tear in the slab, which most likely developed along another Proto-Caribbean fracture zone (e.g. Fz2 Figs. 4 and 8), as the edge of the slab interacted^45^ with the keel of the South American continent.

Low velocities in the mantle wedge are most pronounced above where the reconstruction and kinematic modelling^34^ predict subduction of the Equatorial–Atlantic-Proto-Caribbean boundary (St Lucia to Anguilla, green arrows, Figs. 3a and 6a). Cooper et al.^34^ proposed, from geochemical data, surface-wave tomography, and the distribution of seismicity, that this boundary is particularly hydrated and releases excess fluids into the mantle wedge.

The material subducted along the OAA between ~50 and 35 Ma is visible in the tomography as a high-velocity anomaly between 200 and 450 km depth (Fig. 5b–d). As the large-offset transform, or set of transforms, (labelled as Fz1 in Fig. 4) reached the trench, ocean-floor age at the trench decreased from ~60 to ~40 Myr. The subduction of this much younger lithosphere would have significantly increased the buoyancy of the slab. We propose that this change in buoyancy was responsible for the westward jump of the active arc to the present-day Lesser Antilles Arc at around 30 Ma. The jump would have occurred after enough buoyant lithosphere had subducted to decrease slab pull. This change in buoyancy would have been substantially larger in both magnitude and extent than that of the aseismic Barracuda and Tiburon Ridges (Fig. 1) which are currently at the trench and have previously been proposed to be responsible for the arc jump e.g.^46^. The eastward shift in the arc between 60–50 Ma and 40–30 Ma, while the Grenada back-arc basin opened^11^, is consistent with a lateral shift in the position of the slab around 400 km depth (Figs. 5b, c and 3g and Supplementary Fig. 13), and may require another tear along a fracture zone (Fig. 8).

### Subduction along the Great Arc of the Caribbean (~70–50 Ma, mantle transition zone)

Throughout most of the mantle transition zone, there is a break in the high-velocity anomaly behind the northern part of the current LAA (nG and sG1 in Fig. 5d, e and Supplementary Figs. 12 and 14) which coincides approximately with our predicted location of the ridge subducted between ~50 and 65 Ma. With the resolution gained in VoiLA-P19, it becomes clear (Fig. 6) that this break is not continuous with the shallower low velocities below Martinique, contrary to what was previously proposed^13^, although both features correspond to subducted Proto-Caribbean boundary between North and South America. Note that the Equatorial–Atlantic NAM–SAM transform boundary has only just started subducting and thus does not extend through the slab.

Subduction accelerated from 70 to 50 Ma, when Proto-Caribbean spreading stopped and relatively old lithosphere, up to 80 Myr old, reached the trench (Fig. 4b, c). In Pacific subduction systems, back-arc opening only occurs when older lithosphere is subducted^41,47^. Similarly, in the Proto-Caribbean, it appears that the oldest seafloor exerted sufficient slab pull to open the Yucatán and Venezuelan back-arc basins, and later the Grenada Basin. We propose that this evolution, accompanied by the increasing length of the GAC, led, upon subduction, to the tearing of the Proto-Caribbean lithosphere where it was thinnest, i.e. at the site of the former Proto-Caribbean spreading ridge. Thus, the slab window that originated while the actively spreading ridge subducted could have continued to grow after the extinct ridge subducted (Fig. 8).

The high-velocity anomalies in the transition zone define a much more curved shape than the reconstructed slabs (Fig. 5d, e and Supplementary Figs. 12, 14, 15). In other reconstructions^9,10^, based on ages of arc activity and deformation in the Leeward Antilles, the GAC at 70–50 Ma is strongly curved, bending around the Caribbean plate, partly running parallel to the northwestern margin of South America. We propose that when the late Cretaceous-early Palaeocene arc accreted to South America, part of the slab did as well. That is, the anomaly below the Maracaibo Basin in northwestern Venezuela, labelled sG2 in Fig. 5, is in fact part of the southern GAC slab, sheared off as South and North America started to converge, and the southern end of the subduction zone was forced to bend around South America’s cratonic root. The sG2 seismic velocity anomaly has been previously interpreted to be the result of (ongoing) southward subduction of the southwestern Caribbean^13,22^ and, indeed at lithospheric depths, southward thrusting is evident e.g.^48^. However, the anomaly below 200–250 km has a very steep dip and contains clusters of intermediate-depth earthquakes. Both of these attributes resemble the accreted Carpathian slab below the Vrancea zone in Romania^49^. We propose that most of the anomaly sG2 is a remnant of Proto-Caribbean subduction and only the shallow part is a consequence of recent South America–Caribbean convergence (Fig. 8).

### Subduction along the Great Arc of the Caribbean (~115–80 Ma, shallow lower mantle)

Although embedded in a global inversion to avoid mapping far-field structure into our study region, the VoiLA-P19 model is primarily an improved regional upper-mantle model. For this reason, stages of the evolution predicted by the new plate reconstruction before 60–70 Ma are compared to model UU-P07^31^ which has good lower-mantle resolution below our study area^13^.

The slab that formed during the early phase of GAC subduction is expected to lie in the uppermost lower mantle below northern South America (Fig. 4a, Supplementary Fig. 11 and Supplementary Movie). Van Benthem et al.^13^ associated lower-mantle seismic anomalies below northeast South America with subduction along the southern part of the GAC and a weaker anomaly below Hispaniola (at about 20° north) with subduction along the northern GAC. Our reconstruction shows that both southern and northern Cretaceous GAC slabs are expected to reside below South America, consistent with the fact that the shallow lower-mantle South American velocity anomaly in the UU-P07 model actually consists of two parts (Fig. 7, nG and sG). The single slab dipping from the surface near Hispaniola to 1300–1500 km depth below South America identified by Ren et al.^5^, corresponds to the nG half of the slab (Fig. 8).

The lower-mantle GAC anomalies are clearly distinct from those that previous studies have attributed to Farallon subduction because of continuity with the currently subducting slab below Central and South America (labelled F in Fig. 7)^5,13,50^. The Farallon velocity anomalies have higher amplitudes than the GAC anomalies, consistent with the Farallon slab at this depth having subducted more recently, 50–60 Ma^5^, than our inferred subduction age for the GAC slab at the same depth (90–100 Ma). Higher convergence and sinking rates will lead to a less thermally equilibrated slab and hence a stronger seismic velocity anomaly. Boschman et al.^6^ concluded that Farallon subduction below Central America (Costa Rica) (re)activated as early as 100 Ma. However, apart from the (laterally and vertically) isolated anomaly below Hispaniola, all other lower-mantle anomalies have been attributed to Farallon subduction after 60–70 Ma^5,51^.

The agreement between the tomographic anomalies and projected location of the 100–120-Ma old GAC slab provides supporting evidence for the initiation of westward Proto-Caribbean subduction during the Aptian (~115 Ma) prior to the main phase of CLIP activity, as previously proposed based on geologic and tectonic evidence at the surface^10,12^. The mechanism that would have led to the reversal of the trench and the initiation of GAC subduction of the Proto-Caribbean seafloor with its actively spreading mid-oceanic ridge remains disputed^19,20,52^.

### Evolution of Eastern Caribbean subduction

Our new *P*-wave travel-time tomographic model for the Eastern Caribbean upper mantle and reconstruction of Proto-Caribbean age structure and trench locations through time based on the model of Müller et al.^26^ constrains timings and the likely driving mechanisms of changes in the Great Arc of the Caribbean (GAC) subduction system. Slab fragments below the eastern Caribbean correspond to an accumulation of material (Fig. 8) that was subducted at different trenches at different times but ended up in a similar part of the mantle during the large westward motion of the Americas. The fragmented remnants of this Atlantic subduction system contrast with the large-scale slabs that are imaged below large parts of the Pacific Ring of Fire, e.g., from Farallon subduction. The fragmented slab geometry is likely a consequence of the externally forced subduction of a relatively confined oceanic basin with large buoyancy gradients. The presence of a 90–115-Myr old slab in the shallow lower mantle below north-eastern South America supports the initiation of GAC subduction prior to the most significant phase of Caribbean LIP plume volcanism. In the upper mantle, we find signatures of (1) slab subducted below the Cuban and Aves/Leeward Antilles segments of the GAC around 70–55 Ma, now residing in the mantle transition zone; (2) slab subducted at the Outer Lesser Antilles (including Limestone Caribbees and Virgin Islands) Arc between 55 and 35 Ma, now located between 450 and 250 km depth and (3) slab subducted beneath the present Lesser Antilles to Hispaniola Arc above 250 km depth. Gaps in the slab anomalies coincide with the location of the Proto-Caribbean spreading ridge, a lateral tear below Grenada, and another possible tear in the slab between Hispaniola and Puerto Rico, both probably along Proto-Caribbean fracture zones. Phases of back-arc spreading and trench migration allowed preferential subduction of the oldest parts of the Proto-Caribbean lithosphere, whilst the Oligocene-Miocene advance of the Lesser Antilles Arc resulted from changing slab buoyancy when a large-offset transform was subducted. Thus, our new results demonstrate how the different phases of subduction along the Great Arc of the Caribbean occurred in response to changes in the buoyancy structure of the subducting slab.

## Methods

### Teleseismic tomography

For our tomography, a spherical block parameterisation is used. For the global background model, block size is 5 × 5°. Embedded in this is a finer regional grid covering the area between 90°W and 45°W and between 10°S and 35°N which is divided into 1.5 × 1.5° wide blocks and 14 layers to a depth of 1600 km. The regional grid is further refined (0.75 × 0.75° wide blocks) in the area of interest between 73.5°W and 57°W and between 6.5°N and 23°N (Supplementary Fig. 1 and Table 1).

We inverted a manually-picked set of travel times consisting of 489 measurements recorded at our temporary VoiLA OBS and island station deployment and 3732 registrations from openly available stations in the wider Caribbean (https://www.fdsn.org/webservices/) from regional seismic networks (network codes: 8G, AY, AX, CM, CN, CU, CW, CY, DR, EC, G, GL, IU, JM, LO, MC, MQ, NA, PR, TR, US, VE, WC, WI, XN, XT, ZC*), together with the best quality (iprec = −2 or −3) picks from the EHB catalogue between 1960 and 2016 (^32,53,54^; downloaded from http://www.isc.ac.uk/isc-ehb/ June 8, 2019) (event distribution shown in Supplementary Fig. 2). For this study, only teleseismic rays with epicentral distances >28° and <100° are considered to avoid complexities due to interaction with the crust, mantle discontinuities or core. All details of the data analysis and quality control can be found in Braszus^55^.

To account for uneven data distribution, the EHB dataset with sources or receivers outside the regional grid is clustered into summary rays, such that rays with starting and endpoints within the same 1° × 1° × 50 km cells are averaged. Since the finer parameterisation in the area of interest requires a higher density of rays, no clustering is applied to rays with starting or endpoints located between 110°W– 30°W and 20°S–50°N. The subset of the EHB data with either source or receiver within the local part of the grid comprises 40,926 observations from 215 stations plus 18,734 records from 627 events. Although the EHB catalogue supplies the majority of the data, our manually picked times from 167 events recorded at our 32 OBS and the 192 regional stations provide important complementary coverage as shown in Supplementary Figs. 3 and 9.

Travel times were corrected for elevation, ellipticity and the crustal structure based on CRUST1.0^56^. Because of the distance range used, horizontal ray paths through the crust are avoided and the overall travel time through the partially highly heterogeneous crust is minimised. Crustal corrections were applied to the travel-time residuals to account for the effect of the crust, but additionally we invert for velocities in a shallow layer between 0 and 40 km depth which is able to absorb any further required local deviations from this structure. We do not interpret velocities in this depth layer.

Careful tests of data quality and distribution were done^55^. In the inversion, EHB residuals were included with a weight of 1, while the VoiLA data were weighted depending on their quality with a factor 4 (for A class picks, the uncertainty of ±0.1 s), 2 (for B class, ±0.3 s), 1 (for C class, ±0.5 s) or 0 if rated as poor. After weighting, the VoiLA OBS and regional data contribute 6.6% of the travel times in the local grid. Supplementary Fig. 3 shows that on average, residuals in the study region are slightly fast (with a mean of about −1 s) compared to the global mean and that the patterns in residuals from VoiLA and the local subset of EHB data are consistent, thus showing no indication of systematic biases between the two datasets.

The inversion and raytracing were done using the method from Widiyantoro and Van der Hilst^33^, with a version of the code updated by Weidle^57^. The linearised inversion converges to a stable RMS misfit after 3–4 iterations. Both norm damping, factor λ, and gradient damping, factor γ, are applied. For our preferred model, we choose *λ* = 1.2 m^−1/2^ and *γ* = 1.5 s^−1^ m^−1^, close to the inflection in the trade-off curve between improved misfit (lower residual variance) and increased model complexity (higher model variance) (Supplementary Fig. 4). Small variations in damping parameters yield very similar models. The final preferred tomographic model achieves a residual reduction of 59% with an RMS misfit of 0.790 s.

**Fig. 9:**
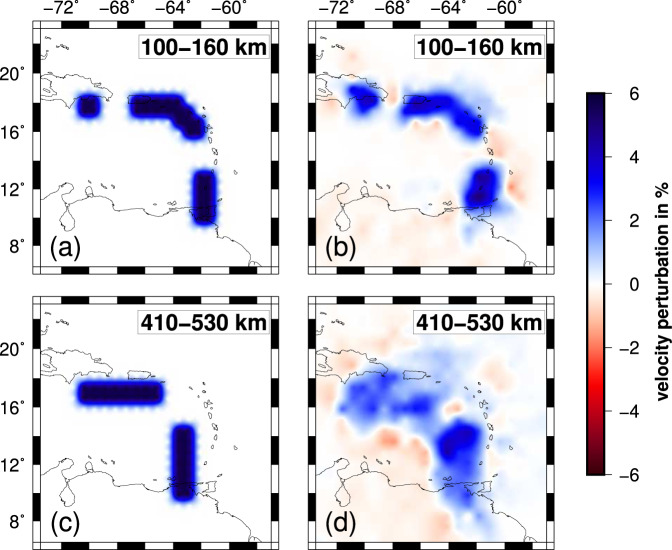
Resolution tests for potential gaps in the slabs. **a**, **b** Input and output in the shallow mantle (local-grid layer 3 between 100 and 160 km depth). **c**, **d** Input and output for the transition zone (local-grid layer 7 between 410 and 530 km depth). For input and output for all layers, see Supplementary Figs. 7 and 8.

Besides the checkerboard tests shown in Fig. 2, Supplementary Figs. 5 and 6, we conducted a characteristic-model resolution test to explore how well previously proposed tears/gaps in the slab can be imaged using our enhanced dataset for the eastern Caribbean. We constructed a synthetic slab model extending from Hispaniola to Trinidad and Tobago based on the results of Van Benthem et al.^13^ and Harris et al.^23^. The dip direction changes from westwards along the Lesser Antilles towards southward beneath Hispaniola and Puerto Rico (Fig. 9 and Supplementary Figs. 7 and 8). The perturbation in *P*-wave velocity is set to +10%. Two 200-km wide gaps in the slab are included in the upper 300 km: one below the central Lesser Antilles and one between Hispaniola and Puerto Rico. For the transition zone, Van Benthem et al.^13^ resolved a slab gap at the bend in the slab below the northern LAA, which we also included in the synthetic model.

As Fig. 9b shows, the general structure of the slab is recovered very well with only minor smearing effects on its edges. The tears in the northwestern and eastern part of the slab are clearly visible. Thus, the previously proposed gaps should be resolved with our new dataset. In the 410-km transition zone, the general shape of the reconstructed slab becomes more blurred as can be seen in Fig. 9d; imaged slab width is about twice the size of the input anomaly. Due to smearing, the gap in the inflection point cannot be resolved with certainty but is clearly imaged as a zone of reduced seismic velocity. Generally, the synthetic tests show that there is sufficient ray coverage in the area of interest to resolve an anomaly of the size and shape of a subducting slab.

### Plate reconstruction adaptations

Our slab geometry predictions are based on the Müller et al.^26^ global plate reconstruction as implemented within the GPlates 2.1 software^58^ (https://www.gplates.org/). This model marks a significant step forward by allowing areas of the otherwise rigid plates to deform during periods of continental extension, collision and shearing^59^. These deformations are parameterised by time and spatially varying meshes that have been built in a systematic manner using a range of geological and geophysical observations. The Gee and Kent^60^ timescale is used throughout. The model also adopts an improved plate-mantle reference frame that is based on a joint inversion of hotspot locations and trails for the past 80 Ma, global trench migration behaviour and estimates of net lithosphere rotation^61^. For our analysis, we made two refinements to the Müller plate boundary geometries as described below. We made no changes to rotation poles.

As a first, and for this paper most important, modification, we refined the position and shape of the Proto-Caribbean mid-ocean ridge axis. This was done by assigning seed points at the continental margins of North and South America prior to rifting at ~150 Ma and then calculating the flowlines as seafloor spreading proceeded. The geometry of the seed points over time was then approximated to give a plate boundary with a minimum number of transform faults between straight mid-ocean ridge segments (Fz1-3, Fig. 4). Owing to the shape of the Bahamas Bank–Demerara Plateau part of the conjugate continental margins, longer offset transform faults were needed in the east than in the west. The geometry of the plate boundary between North America and Africa that opened during this time (the seafloor produced being still preserved in the Central Atlantic) was left unchanged.

**Fig. 10:**
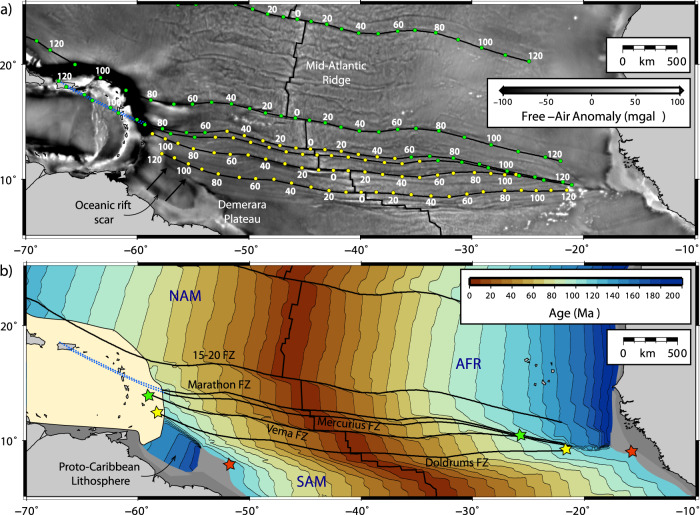
Present-day age structure of the Central Atlantic predicted by the plate reconstruction by Müller et al.^26^ with our plate boundary adjustments. **a** Satellite-derived free air-gravity anomaly, with observed fracture zone traces (thick black lines). Coloured dots (ages in Ma) are from the plate reconstruction predictions, with green dots representing segments produced by Central/ Equatorial–Atlantic spreading (NAM–AFR poles), and yellow dots those produced by Equatorial–Atlantic spreading (SAM–AFR poles). See Supplementary Fig. 10 for details. A northward propagation of NAM–SAM–AFR triple junction is shown by the transfer along Mercurius and Marathon FZs from NAM to SAM poles. In other words, the NAM–SAM boundary has not been stable over time, especially for the older parts of the oceanic lithosphere relevant for our study. **b** Predicted seafloor age structure. Colour scale from ref. ^68^. Green, yellow and red stars indicate matching points along the conjugate margins. Note the small fragment of relatively old Proto-Caribbean lithosphere entering the southern Lesser Antilles. Dotted blue line illustrates the trace of the subducted Marathon and Mercurius fracture zones, which are interpreted to form the boundary between the Equatorial and Central Atlantic lithosphere.

The second aspect we modified was the geometry of the boundary between the Proto-Caribbean/Central-Atlantic opening and the Equatorial–Atlantic opening. A source of uncertainty in the plate reconstruction is the location of the North and South American plate boundary today and in the past. Currently available earthquake and geodetic data show the boundary to be diffuse^62^, with most workers placing it at the Fifteen-Twenty Fracture Zone (Fig. 10). However, based on fracture zone geometries, Müller and Roest^63^ suggested that the plate boundary moved northwards to its present position during the Cenozoic. To test this idea, we computed synthetic flowlines within GPlates using combinations of North-West African (AFR) and North/South American (NAM/SAM) rotation poles see also^64^ and compared them to the fracture zone traces observed from modern satellite altimetry data (^65^; see coloured circles in Fig. 10, Supplementary Fig. 10). The fracture zone traces on the oldest part of the African plate were especially important in the analysis as the equivalent ones on the American side have been subducted. We found that the older parts of both Mercurius and Marathon Fracture Zones were best matched with NAM–AFR poles, and the younger parts SAM–AFR poles, such that the AFR-NAM–SAM Triple Junction passed the Mercurius Fracture Zone at 60 Ma and Marathon Fracture Zone at 50 Ma (Supplementary Fig. 10). The fracture zones south of, and including, Vema are entirely within the Equatorial–Atlantic domain. In the Müller et al.^26^ model, the northward propagating Equatorial–Atlantic ridge axis intersects the Proto-Caribbean/Atlantic ridge north of the Marathon Fracture Zone. However, we demonstrate that the geometry of this fracture zone can be explained entirely by relative NAM–AFR motion prior to ~60 Ma. To honour this observation, we assigned the Mercurius Fracture zone as the intersection point (marked with a yellow star in Fig. 4). We, therefore, made this small adjustment to the plate boundary geometries for this time period.

Since 60 Ma, there has been a small amount of compression across the Equatorial–Atlantic NAM–SAM boundary which has been accommodated along a diffuse zone between the Mid-Atlantic Ridge and the Lesser Antilles trench forming features such as the Barracuda and Tiburon Ridges (Fig. 1). The Atlantic NAM–SAM transform plate boundary currently has very little relative motion, with the two Americas moving away from Africa effectively as one.

The present-day locations of the slabs subducted at the GAC, OAA and LAA are predicted by capturing the position of the GAC trench system through time, starting from the initial position of the westward facing trench at 120 Ma, and moving them according to one of the three scenarios (Supplementary Fig. 11), vertical sinking (case 1), motion with North or South America (case 2) or a hybrid scenario (case 3). The trench position is defined at a location 150 km west of the leading edge of the plate, a distance similar to the present-day distance between the front of the accretionary prism and the actual trench. Comparison of vertical and hybrid trench positions with model VoiLA-P19 and UU-P07 are shown in Supplementary Figs. 12, 14–16.

### *Network references

**8G**: Meltzer, A., & Beck, S. (2016). 2016 Pedernales Earthquake Aftershock Deployment Ecuador [Data set]. *International Federation of Digital Seismograph Network*s, doi: 10.7914/SN/8G_2016; **CM**: Servicio Geologico Colombiano. (1993). *Red Sismologica Nacional de Colombia* [Data set]. International Federation of Digital Seismograph Networks, doi: 10.7914/SN/CM; **CN**: Geological Survey of Canada. (1989). Canadian National Seismograph Network. International Federation of Digital Seismograph Networks, doi: 10.7914/SN/CN; **CU**: Albuquerque Seismological Laboratory (ASL)/USGS. (2006). *Caribbean USGS Network*. International Federation of Digital Seismograph Networks, doi10.7914/SN/CU; **CW**: National Centre for Seismological Research (CENAIS Cuba). (1998). *Servicio Sismologico Nacional de Cuba*. International Federation of Digital Seismograph Networks, doi: 10.7914/SN/CW; **DR**: National Seismological Centre Of Autonomous University of Santo Domingo. (1998). *CNS-UASD* [Data set]. International Federation of Digital Seismograph Networks, doi: 10.7914/SN/DR; **IU**: Albuquerque Seismological Laboratory (ASL)/USGS. (1988). Global Seismograph Network (GSN - IRIS/USGS). International Federation of Digital Seismograph Networks. doi: 10.7914/SN/IU; **G**: GEOSCOPE - French Global Network of broadband seismic stations. Institut de Physique du Globe de Paris & Ecole et Observatoire des Sciences de la Terre de Strasbourg (EOST) - doi:10.18715/GEOSCOPE.G; **LO**: Instituto Politecnico Loyola. (2012). Observatorio Sismológico Politécnico Loyola. International Federation of Digital Seismograph Networks, doi: 10.7914/SN/LO; **NA**: KNMI. (2006). *Caribbean Netherlands Seismic Network*. Royal Netherlands Meteorological Institute (KNMI), doi: 10.21944/dffa7a3f-7e3a-3b33-a436-516a01b6af3f; **PR**: University of Puerto Rico. (1986). *Puerto Rico Seismic Network (PRSN) & Puerto Rico Strong Motion Program (PRSMP)*. International Federation of Digital Seismograph Networks, doi: 10.7914/SN/PR; **TR**: Albuquerque Seismological Laboratory (ASL)/USGS. (2006). *Caribbean USGS Network*. International Federation of Digital Seismograph Networks, doi: 10.7914/SN/CU; **US**: Albuquerque Seismological Laboratory (ASL)/USGS. (1990). *United States National Seismic Network*. International Federation of Digital Seismograph Networks, doi: 10.7914/SN/US; **VE**: Fundación Venezolana De Investigaciones Sismológicas (FUNVISIS), Caracas. (2000). *Red Sismológica Satelital Nacional* [Data set]. International Federation of Digital Seismograph Networks, doi: 10.7914/SN/VE. **WI**: Institut De Physique Du Globe De Paris-IPGP. (2008). *GNSS, seismic broadband and strong motion permanent networks in West Indies*. Institut de Physique du Globe de Paris-IPGP, doi: 10.18715/antilles.WI; **XN**: Levander, A. (2008). *Bolivar: Western Venezuela*. International Federation of Digital Seismograph Networks, doi: 10.7914/SN/XN_2008; **XT**: Terry Wallace, F. V. (2003). Crust-Mantle Interactions during Continental Growth and High-Pressure Rock Exhumation at an Oblique Arc-Continent Collision Zone: SE Caribbean Margin. International Federation of Digital Seismograph Networks. doi: 10.7914/SN/XT_2003; **ZC**: Pulliam, J. (2013). Greater Antilles Seismic Program. International Federation of Digital Seismograph Networks. 10.7914/SN/ZC_2013.

## Supplementary information

Supplementary Information

Description of Additional Supplementary Files

Caribbean Reconstruction Movie

## Data Availability

The global travel-time catalogue was downloaded from the International Seismological Centre (http://www.isc.ac.uk/isc-ehb/). Data from the regional networks were obtained from IRIS-DMC (the Incorporated Research Institutions for Seismology Data Management Center, http://ds.iris.edu/ds/nodes/dmc/forms/breqfast-request/), from where the broadband OBS data collected in the VoiLA project will also be available by the end of the project embargo, April 2021. Braszus^55^ includes a list of the regional events and stations used. Model VoiLA-P19 is available at doi:10.5445/IR/1000130417. The GPlates files for our updates to the Müller et al.^26^ reconstruction are in the Supplementary Material. Source data are provided with this paper.

## Source data

Source Data

## Code availability

The seismic tomography code is available on request from Prof. Andreas Rietbrock (Karlsruhe Institute of Technology).
