# Supplementary material for: Subduction history of the Caribbean from upper-mantle seismic imaging and plate reconstruction
Source: Nat Commun. 2021 Jul 9;12:4211. doi: 10.1038/s41467-021-24413-0 (PMC8270990; doi:10.1038/s41467-021-24413-0)
Supplement: Supplementary file 1 — Supplementary Information [file 41467_2021_24413_MOESM1_ESM.docx]

**Supplementary material**

Braszus et al. Nat. Comms. 2021

**Subduction history of the Caribbean from upper-mantle seismic imaging and plate reconstruction**

This supplement provides further supporting material for the following:

**Note S1.** Teleseismic tomography method and resolution ………………………………..pg 1

**Note S2.** Reconstruction of plate domains and slab positions at depth……………..pg 9

**Note S3.** Comparison tomography and reconstructed slab positions……………….. pg 11

**Supplementary Note S1. Teleseismic tomography method and resolution**

**
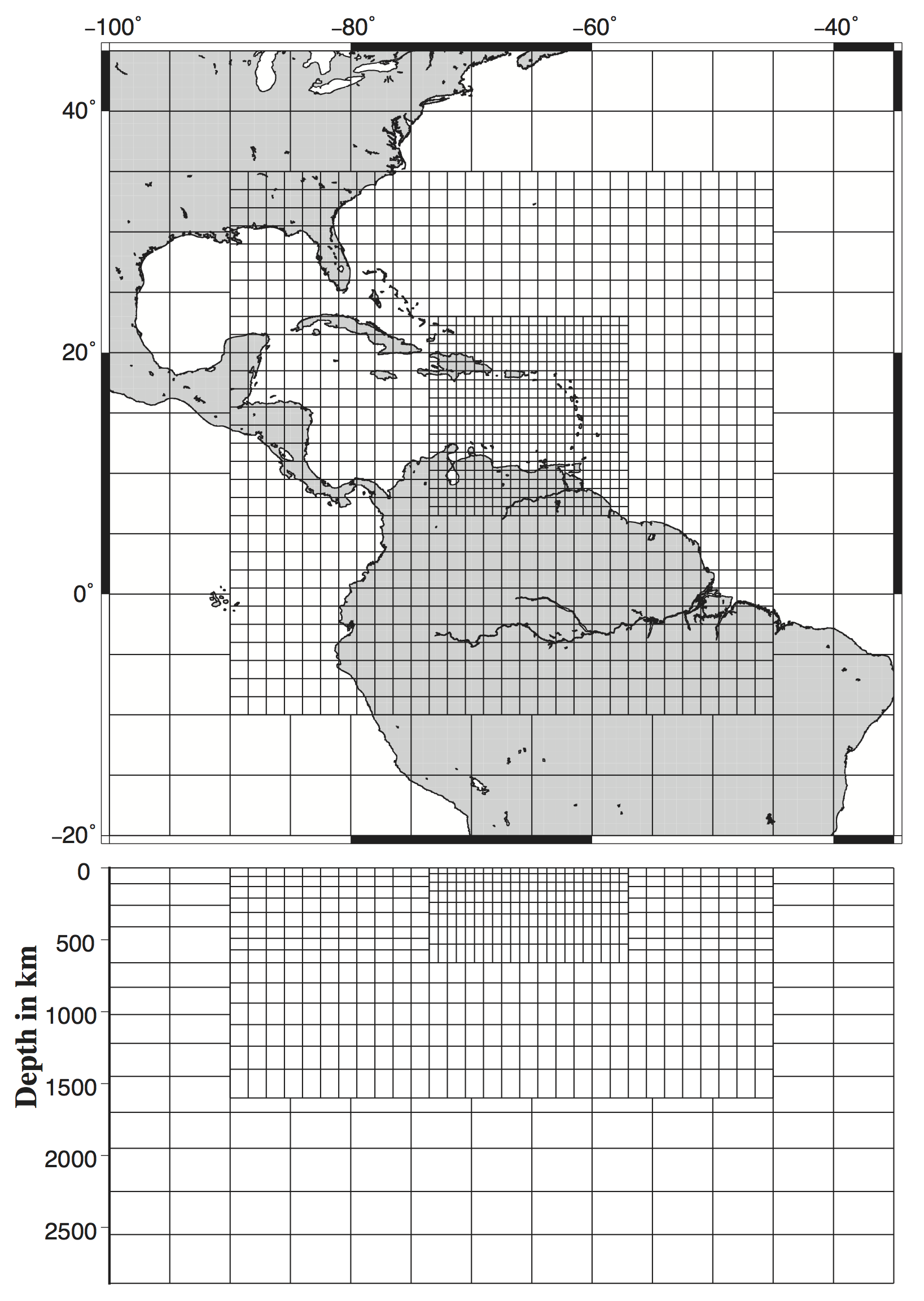
**

**Supplementary Figure 1.** **Tomography model grid.** Horizontal section (top) and vertical section (bottom) illustrating the grid used in the tomographic inversion, where regional and local grids were embedded into a coarser global grid. See Supplementary Table 1 for layer depths, cell sizes and reference velocities.

|  | **Local grid** | | **Regional grid** | | **Global grid** | |
| --- | --- | --- | --- | --- | --- | --- |
| Layer no. | Layer depths (km) | V_P_ (km/s) | Layer depths (km) | V_P_ (km/s) | Layer depths (km) | V_P_ (km/s) |
| *1* | *0-40* | *6.34* | *0-60* | *6.91* | *0-110* | *7.43* |
| *2* | *40-100* | *8.04* | *60-130* | *8.05* | *110-260* | *8.22* |
| *3* | *100-160* | *8.07* | *130-210* | *8.17* | *260-410* | *8.73* |
| *4* | *160-240* | *8.25* | *210-310* | *8.46* | *410-660* | *9.76* |
| *5* | *240-320* | *8.53* | *310-410* | *8.82* | *660-830* | *10.98* |
| *6* | *320-410* | *8.84* | *410-490* | *9.47* | *830-1020* | *11.32* |
| *7* | *410-530* | *9.54* | *490-570* | *9.74* | *1020-1220* | *11.63* |
| *8* | *530-660* | *9.96* | *570-660* | *10.02* | *1220-1450* | *11.95* |
| *9* |  |  | *660-800* | *10.95* | *1450-1700* | *12.27* |
| *10* |  |  | *800-940* | *11.23* | *1700-1950* | *12.58* |
| *11* |  |  | *940-1090* | *11.47* | *1950-2250* | *12.90* |
| *12* |  |  | *1090-1240* | *11.70* | *2250-2550* | *13.24* |
| *13* |  |  | *1240-1400* | *11.93* | *2550-2889* | *13.58* |
| *14* |  |  | *1400-1600* | *12.18* |  |  |
| Horizontal spacing | *0.75°x0.75°* | | *1.5°x1.5°* | | *5°x5°* | |

**Supplementary Table 1.** **Layer depths and P-wave velocities (V_P_) of the tomography starting model.** Parameters are given for the global, regional and local parameterization. The initial velocity of a layer is calculated as the weighted average of velocities from the corresponding depths in the AK135 reference model ^37^.

**Supplementary Figure** **2.** **Distribution of seismic events for model VoiLA-P19.** (a) Distribution of stations (blue) and events (red) from the EHB catalogue ([www.isc.ac.uk/isc-ehb](http://www.isc.ac.uk/isc-ehb), accessed June 2019) after clustering and filtering out low quality picks as well as phases with epicentral distances < 28^o^. (b) Events from the VoiLA data set (167 in total). The number of records per event is indicated by the colour code. Concentric circles mark the epicentral distance to the centre of the network at 14°N and 61°W

| **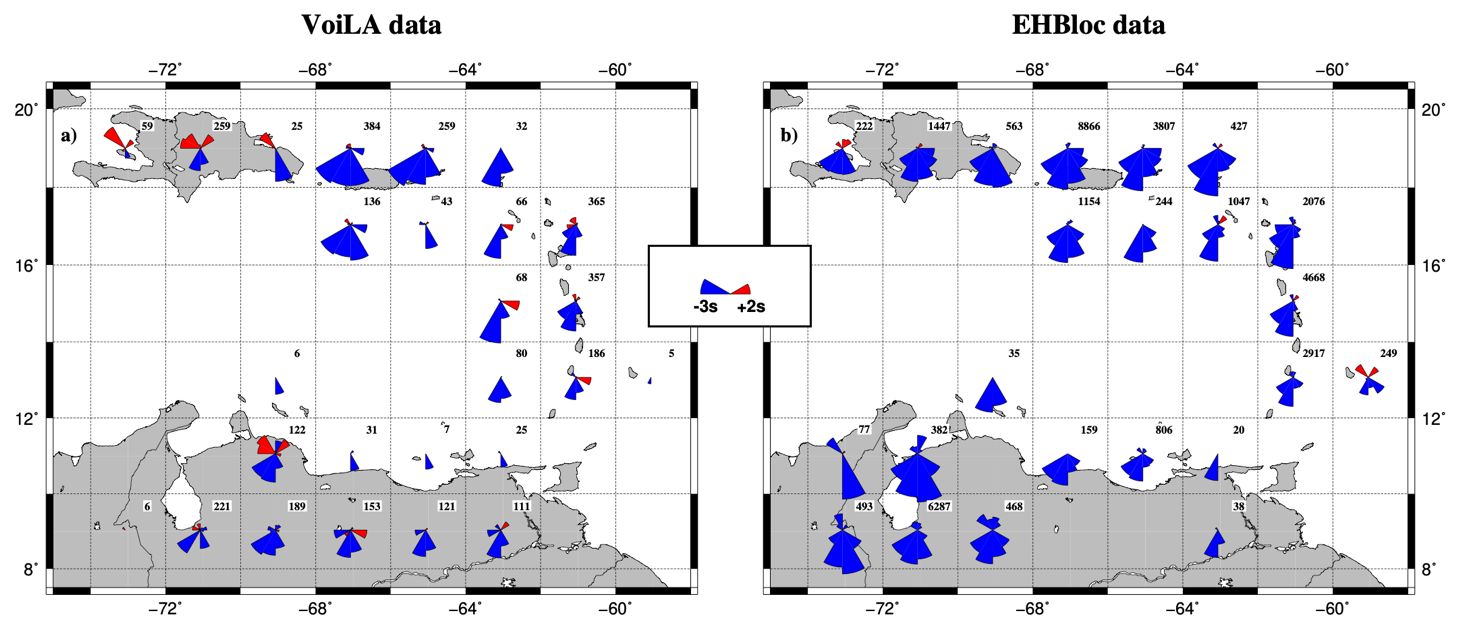** |
| --- |
| **Supplementary Figure 3. P-wave travel-time residuals as a function of back-azimuth.** Residuals for VoiLA data (a) and the local subset of the EHB data (b). Blue colours indicate residuals that are fast compared to the global mean, red colours slow residuals. The number of observed arrivals in each 1°x1° cell is indicated in the upper right corner of the rose diagram. |


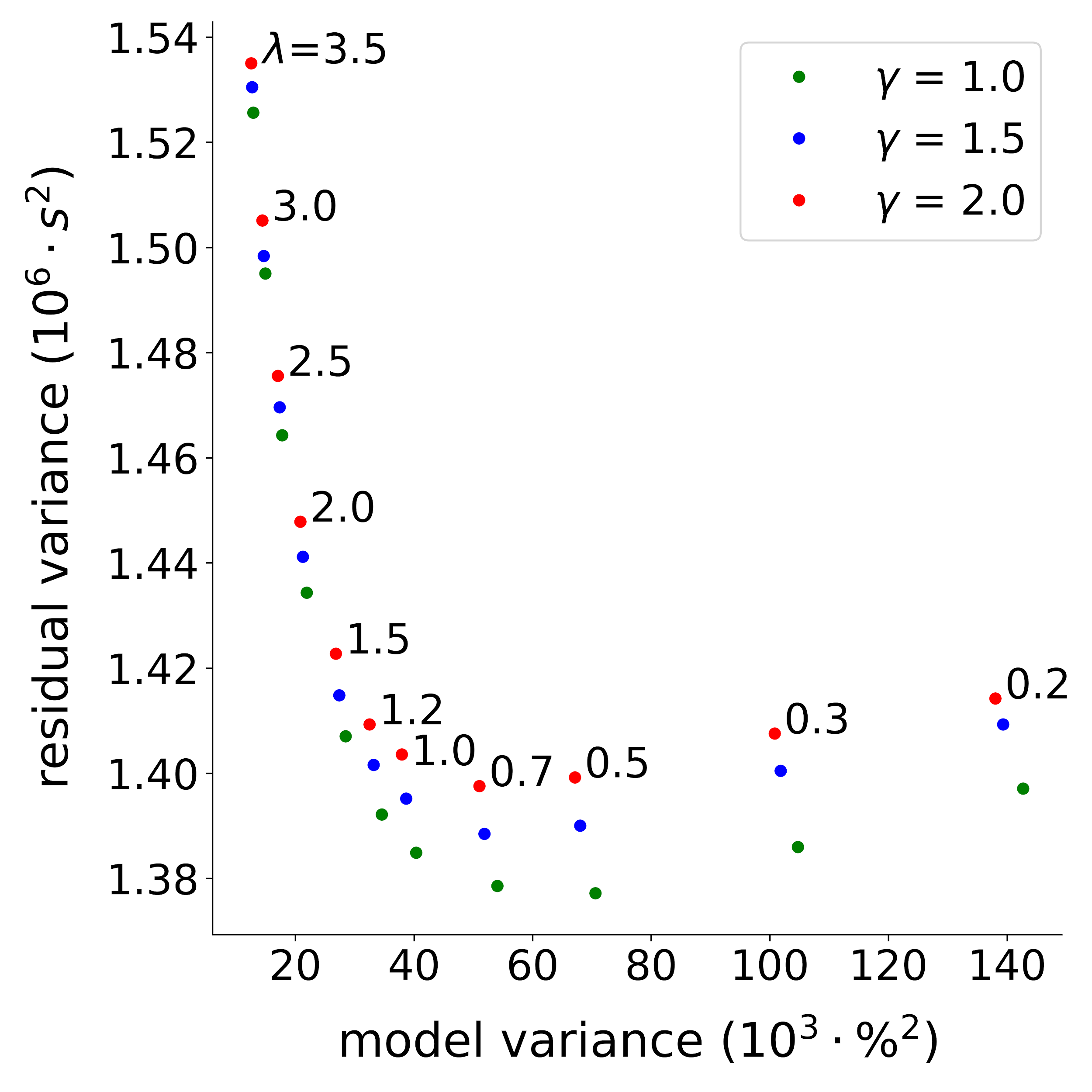


**Supplementary Figure 4.** **Trade-off between residual variance and model variance after one iteration.** Variance is shown for selected combinations of norm damping λ (value labeled on each branch, in units of m^-1/2^) and gradient damping γ (color-coded, in units s^-1^m^-1^). Preferred model has λ=1.2 and γ=1.5.

***
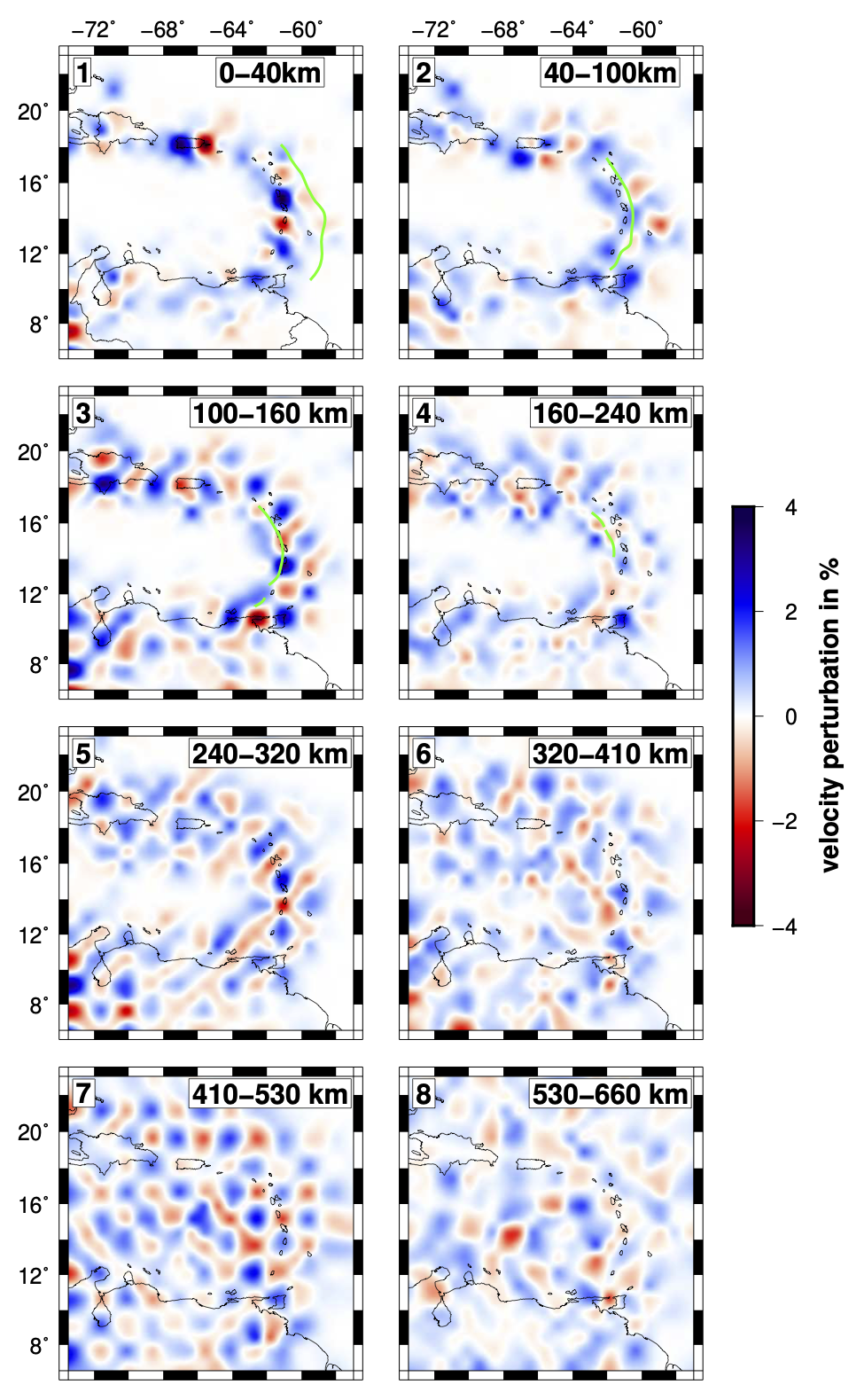
***

**Supplementary Figure 5.** **VoiLA-P19 resolution illustrated by checkerboard tests.** The local-grid input model consists of checkerboard shaped anomalies in the odd numbered layers and no anomalies in even numbered layers. The horizontal distance between positive and negative anomalies of ±10% is 1.5^o^ and their signs are reversed in every perturbed layer. Results shown for all local grid layers illustrate lateral resolution in each of the odd layers as well as smearing into originally zero-anomaly even layers. Green contours on the top four panels show slab position based on seismicity [Bie et al., 2020].

***
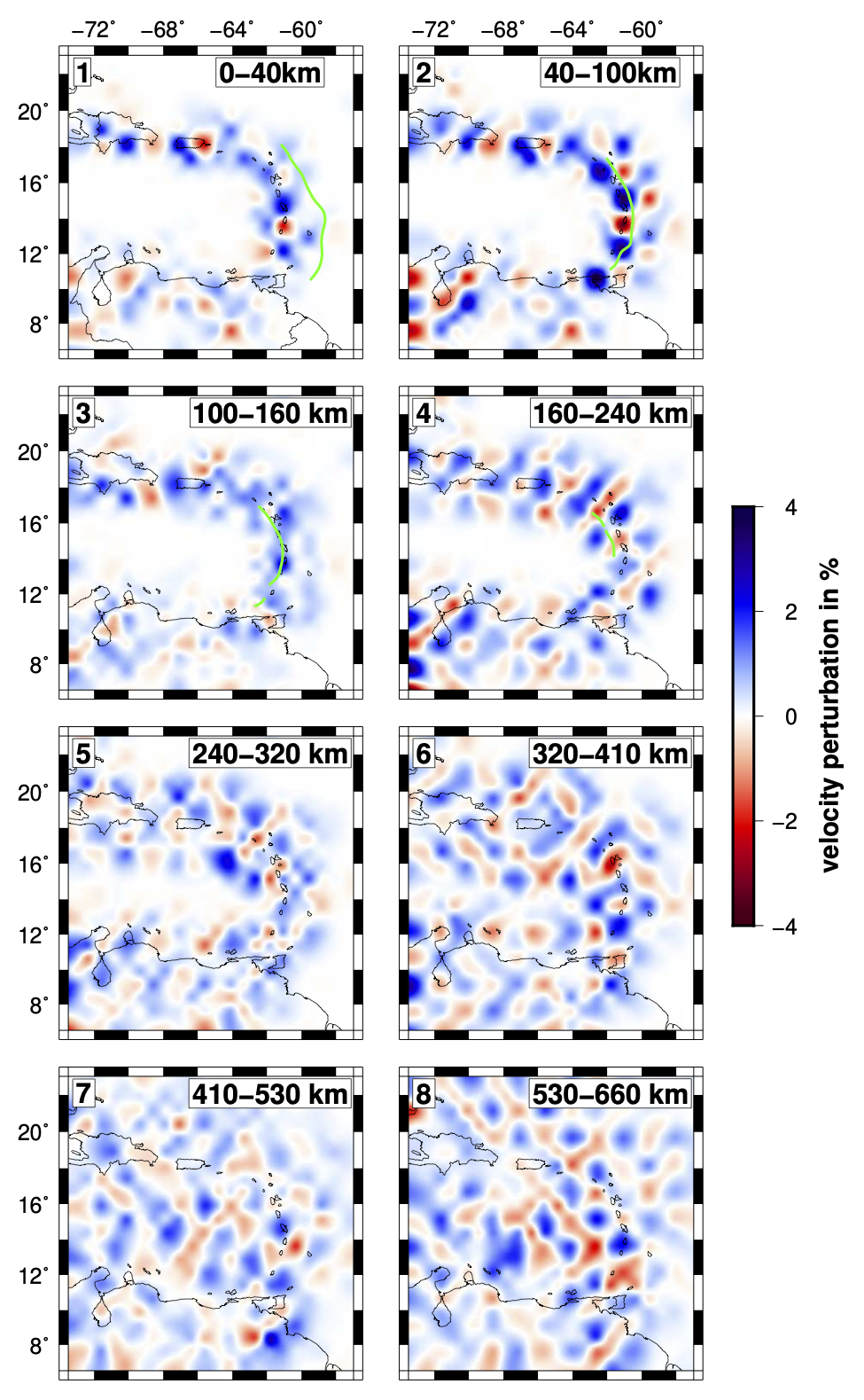
***

**Supplementary Figure 6.** **VoiLA-P19 resolution illustrated by checkboard tests.** Same as Supplementary Figure 5, but with input checkers placed in the even numbered layers of the local grid and no input anomalies in the odd-numbered layers.

***
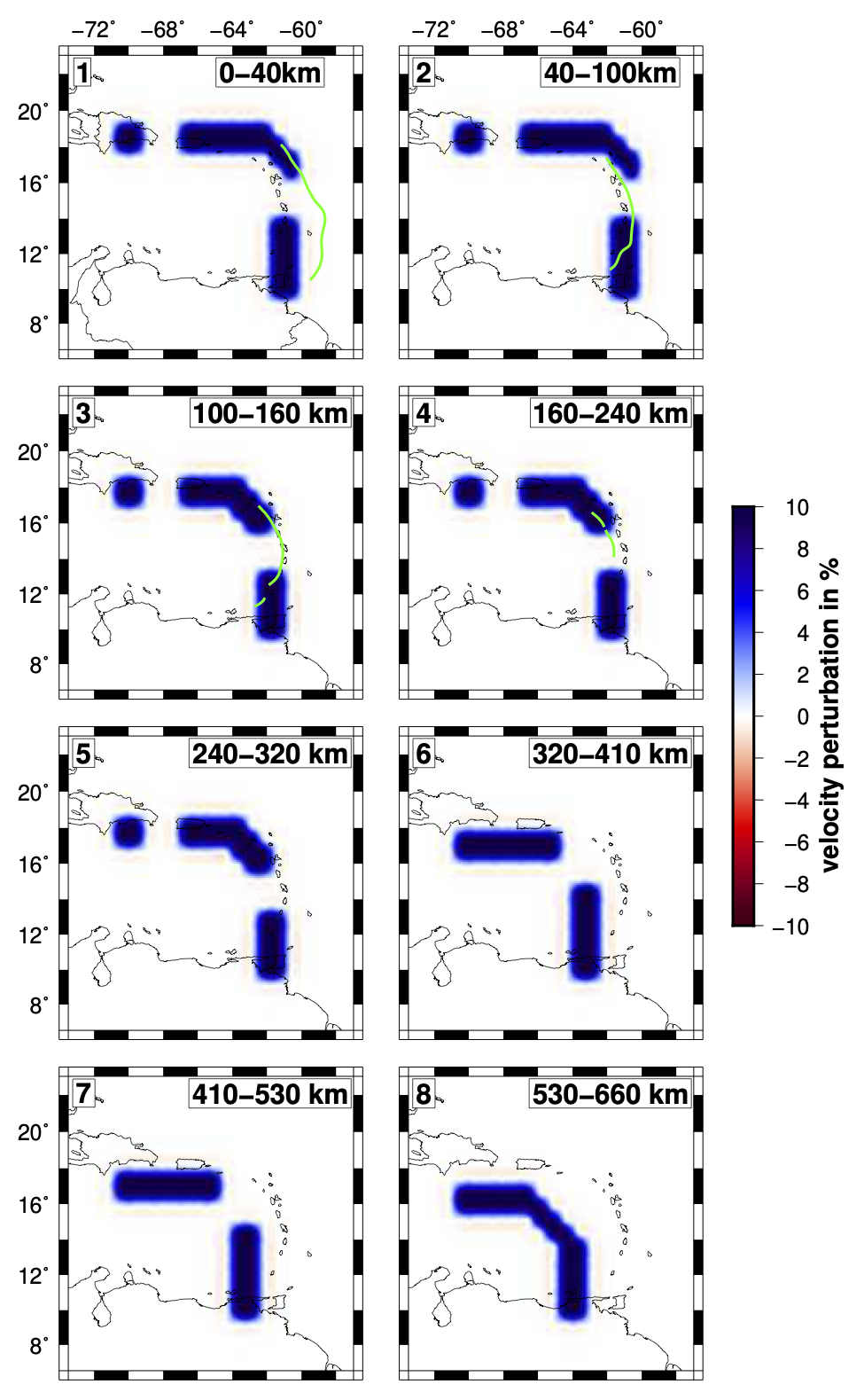
***

**Supplementary Figure 7.** **Synthetic slab models for resolution test.** Input structures for resolution test of synthetic +10% slab velocity anomalies including previously proposed gaps. All layers of the local grid where slab anomalies were input are shown. Green contours on the top four panels show slab position based on seismicity [Bie et al., 2020].


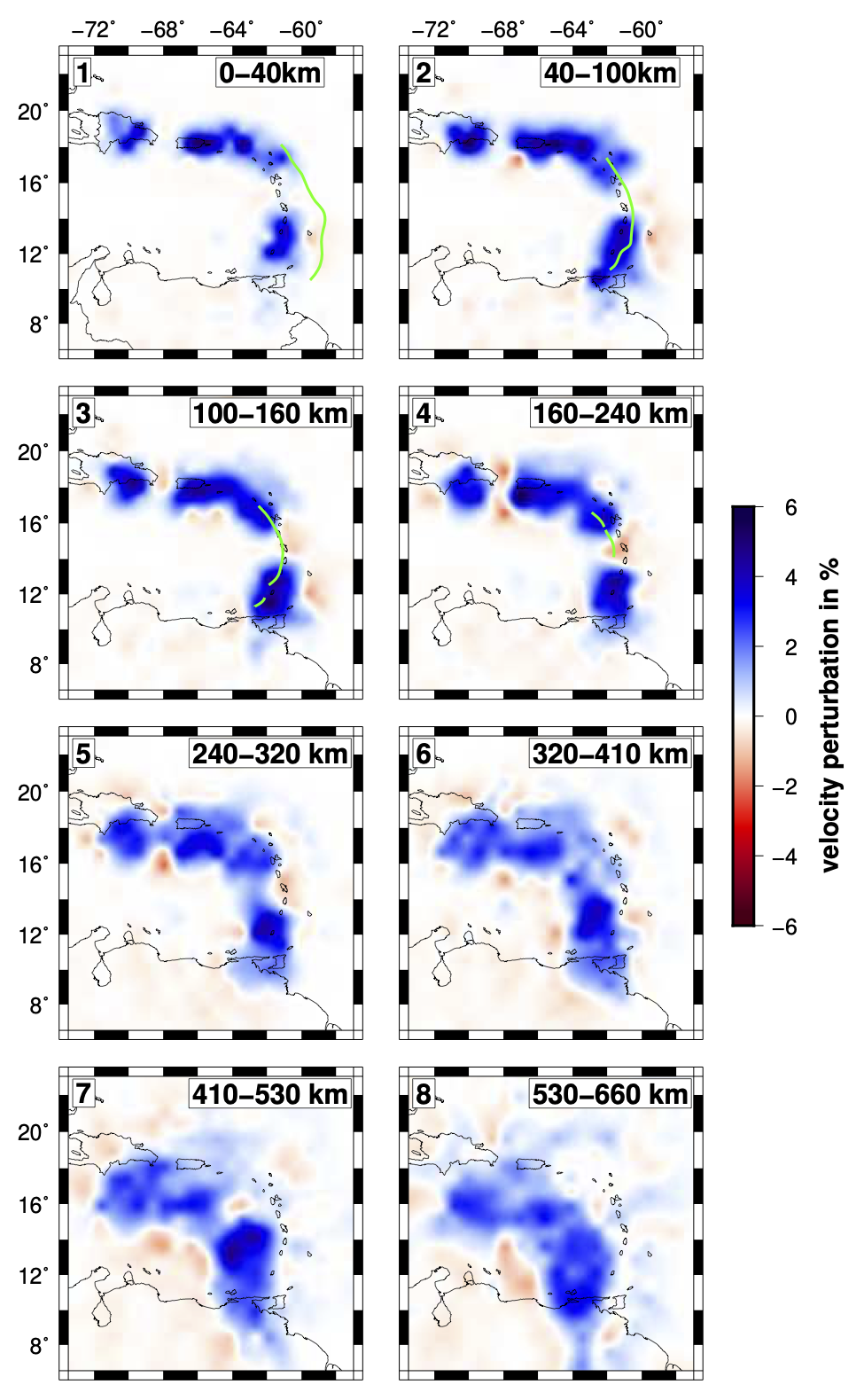


**Supplementary Figure 8.** **VoiLA-P19 resolution of synthetic slab models.** Recovery of the synthetic slab anomalies in Supplementary Figure 7 in the tomographic inversion for the same distribution of sources-receivers as the combined EHB and VoiLA data sets, for all layers of the local grid.

*
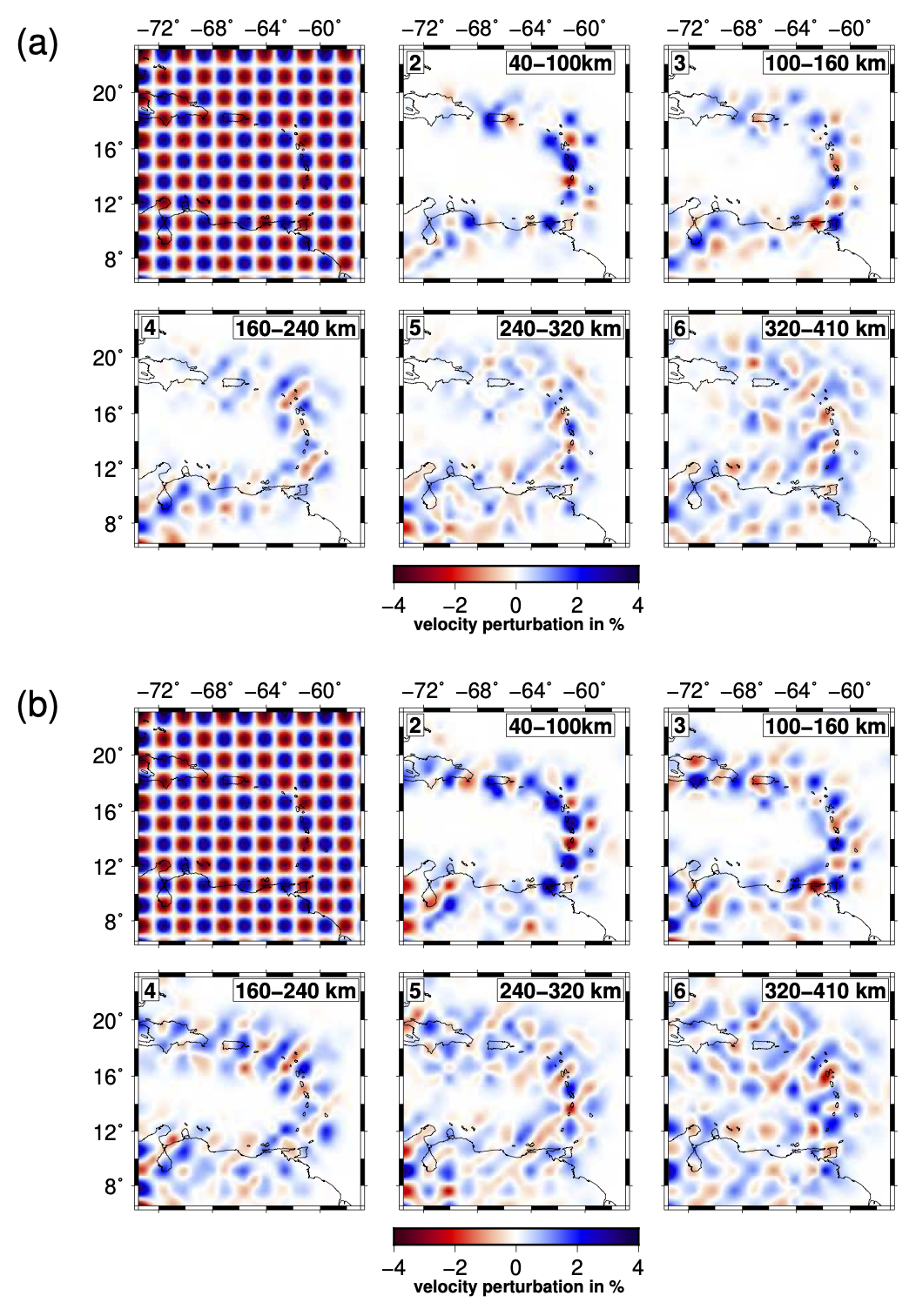
*

**Supplementary Figure 9. Improvement in resolution with our data set** **compared to 2004 data used in Amaru**^33^. (a) Checkerboard test using data from EHB catalogue until 2004. (b) Same test using the EHB catalogue until 2016, data from regional networks and the VoiLA deployment. In each panel, the input model (alternating positive and negative velocity perturbations of 10% either in the odd-numbered or even-numbered layers of the local grid) is shown in the top left. The horizontal distance between positive and negative anomalies is 1.5^O^ and their signs are reversed in every perturbed layer. Examples are shown of recovery in layer 2, layer 4, and layer 6 for a test where input anomalies are placed only in even-numbered layers. Recoveries in layer 3 and layer 5 are illustrated for input anomalies in odd-numbered layers.

**Supplementary Note S2. Reconstruction of plate domains and slab positions at depth**

***
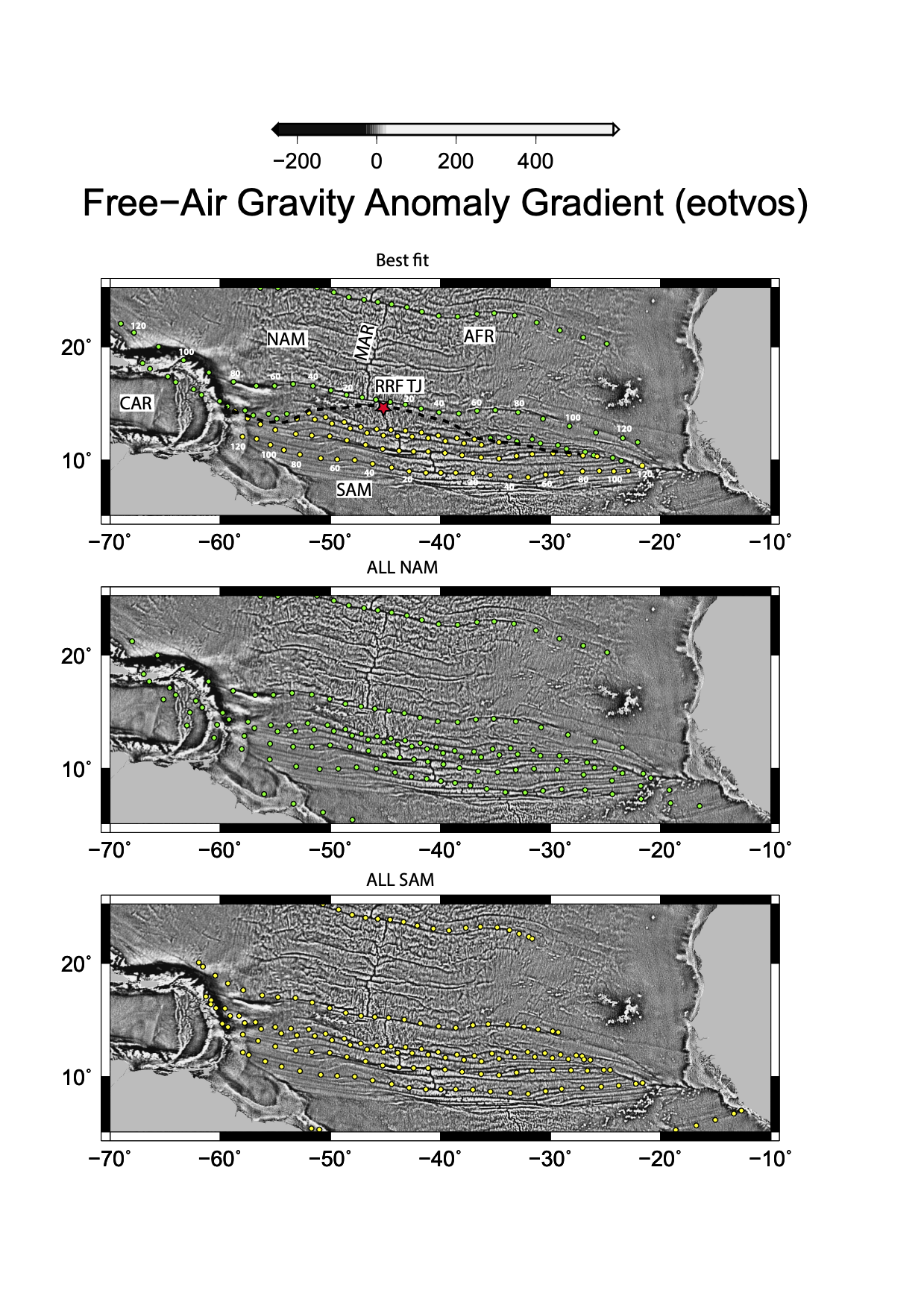
***

**Supplementary Figure 10.** **Satellite-derived free-air gravity anomaly, with illustration of flow line modelling.** Coloured dots (ages in Ma) are from the plate reconstruction predictions, with green dots representing segments produced by Central/ Equatorial Atlantic spreading (NAM-AFR poles), and yellow dots those produced by Equatorial Atlantic spreading (SAM-AFR poles). The migration of the NAM-SAM-AFR ridge-ridge-transform triple junction (RRFTJ) is marked on the top panel with a bold dashed black line. MAR – Mid-Atlantic Ridge

**See Supplementary Movie of slab positions for case (1) – vertical sinking**

**Supplementary Figure 11** **Predicted slab positions** **for three slab motion cases**. (a) Case 1 - The slabs sink vertically, i.e. move in a mantle reference frame. (b) Case 2 - The slabs move as if attached to North and South America (depending on whether subducted north or south of the Proto-Caribbean ridge). (c) Case 3 - A hybrid scenario, where slabs are attached to North or South America until Cuba fully separates by 50 Ma, and afterwards already subducted slabs move in a mantle reference frame. Plate motions are from Müller et al. [2019]. The present-day locations of the slabs subducted at the GAC, OAA and LAA are predicted by capturing the position of the Caribbean trench through time, where the trench position is defined at a location 150 km west of the leading edge of the plate, a distance similar to the present-day distance between the front of the accretionary prism and the actual trench. Slab positions shown every 10 million years (subduction times in red numbers), with in yellow and green respectively, material formed north and south of the Proto- Caribbean spreading ridge (star when active, diamond when extinct upon subduction). Oldest position marks the trench at GAC initiation between 120-110 Ma.

**Supplementary Note S3. Comparison tomography and reconstructed slab positions**

***
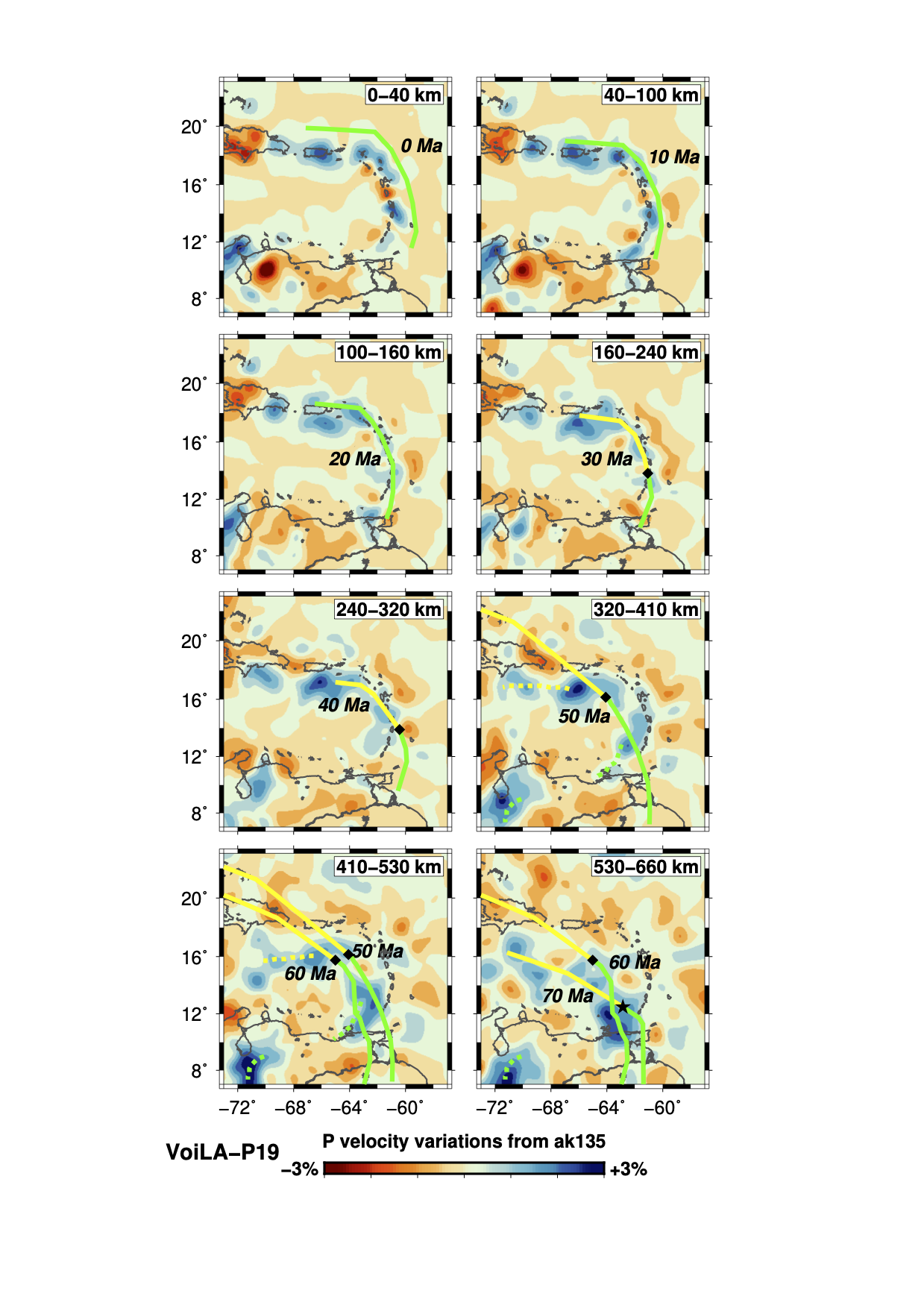
***

**Supplementary Figure** **12**. **VoiLA-P19 P-velocities in the upper mantle with predicted slab positions from case 1.** Velocity anomalies relative to global model AK135 ^37^ are shown at all local grid depth levels. Superimposed are the reconstructed positions of the slabs assuming vertical sinking (case 1), in coloured lines labelled with the time of subduction. At 50-70 Ma: yellow lines: slab subducted at the Cuban part of the GAC, green lines: slab subducted at the Aves/Leeward Antilles part of the GAC, with the black diamond marking the location of the spreading centre if extinct, black star the location of the spreading centre if active at the time of subduction. At 0-50 Ma, green lines denote the position of slabs subducted along the Outer Antilles Arc (at 30-50 Ma) or the Lesser Antilles Arc (at 0-20 Ma). Dotted lines indicate possible post-subduction deformation of the slab.

***
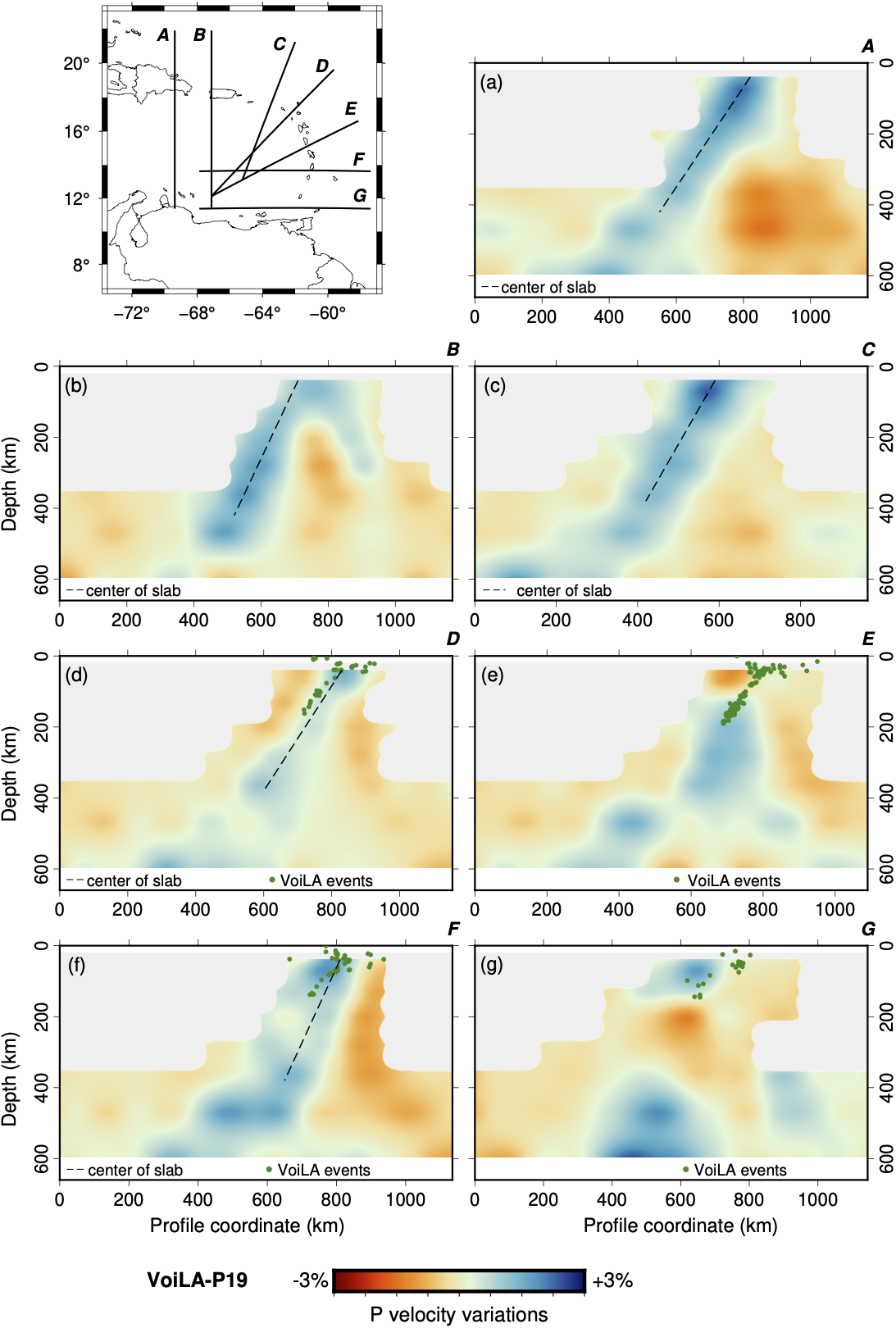
***

**Supplementary Figure** **13** **Vertical cross sections through model VoiLA-P19.** Locations of the cross sections are shown on the map at the top left. Colour scale is the same as in Supplementary Figure 12. Cross sections B, C, E and G are also shown in Figure 3. Structures are masked where resolution is limited, i.e. at depths less than 300 km above and behind the slab. Seismicity shown with green dots is from the relocated VoiLA data set ^31^. Dashed line marks the estimated centre of the slab.

***
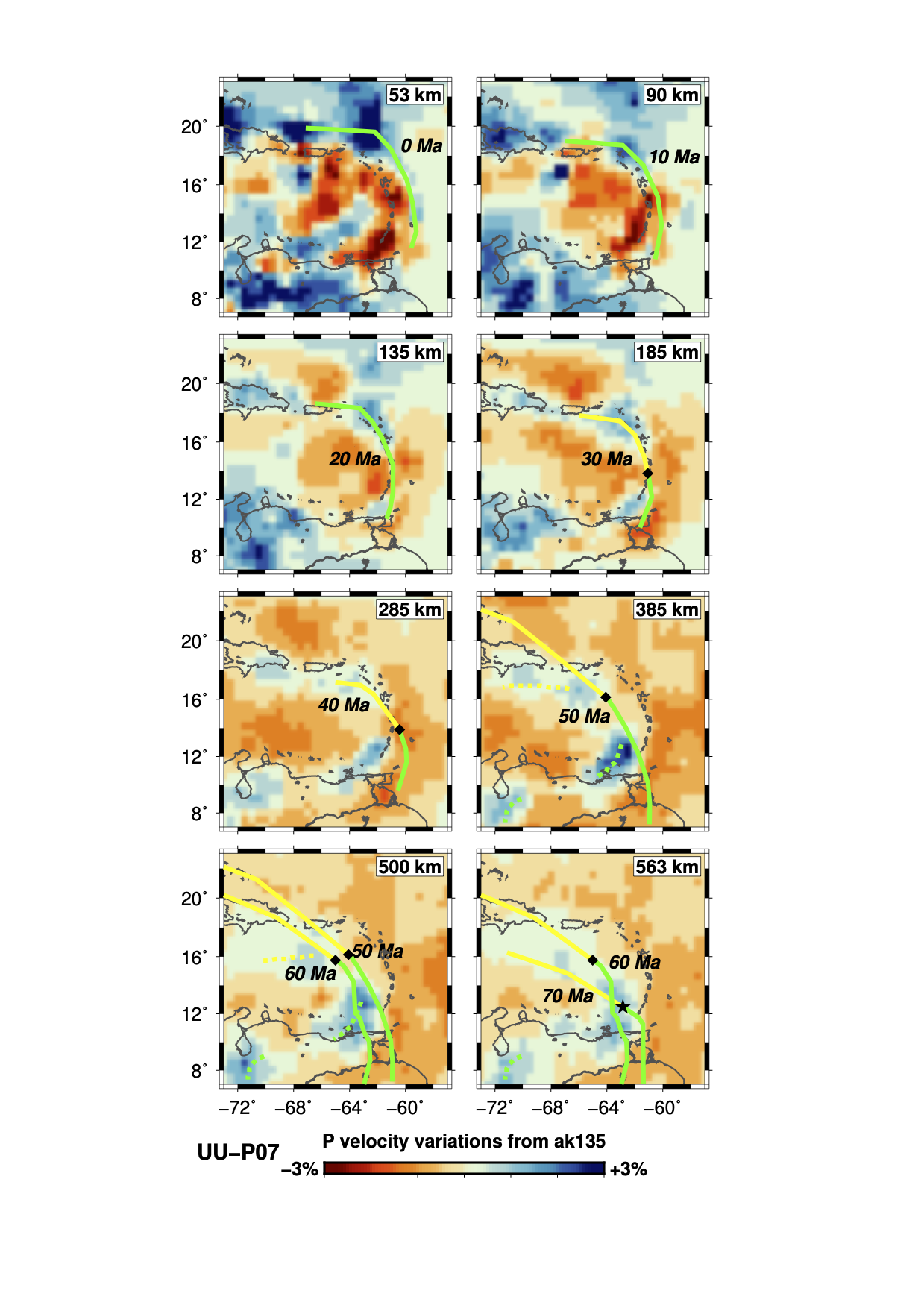
***

**Supplementary Figure** **14.**  **Model UU-P09 P-velocities** ^33,13^ **in the upper mantle with predicted slab positions from case 1.** Velocity anomalies are relative to global model AK135 ^37^. Depths are comparable to those shown for model VoiLA-P19 in Supplementary Figure 12. Superimposed are the reconstructed slab positions assuming vertical sinking (case 1), in coloured lines labelled with the time of subduction. At 60-70 Ma: yellow lines: slab subducted at the Cuban part of the GAC, green lines: slab subducted at the Aves/Leeward Antilles part of the GAC, with the black diamond marking the location of the spreading centre if extinct, black star the location of the spreading centre if active at the time of subduction. At 0-50 Ma, green lines denote the position of slabs subducted along the Outer Antilles Arc (at 30-50 Ma) or the Lesser Antilles Arc (at 0-20 Ma). Dotted lines indicate possible post-subduction deformation of the slab.

***
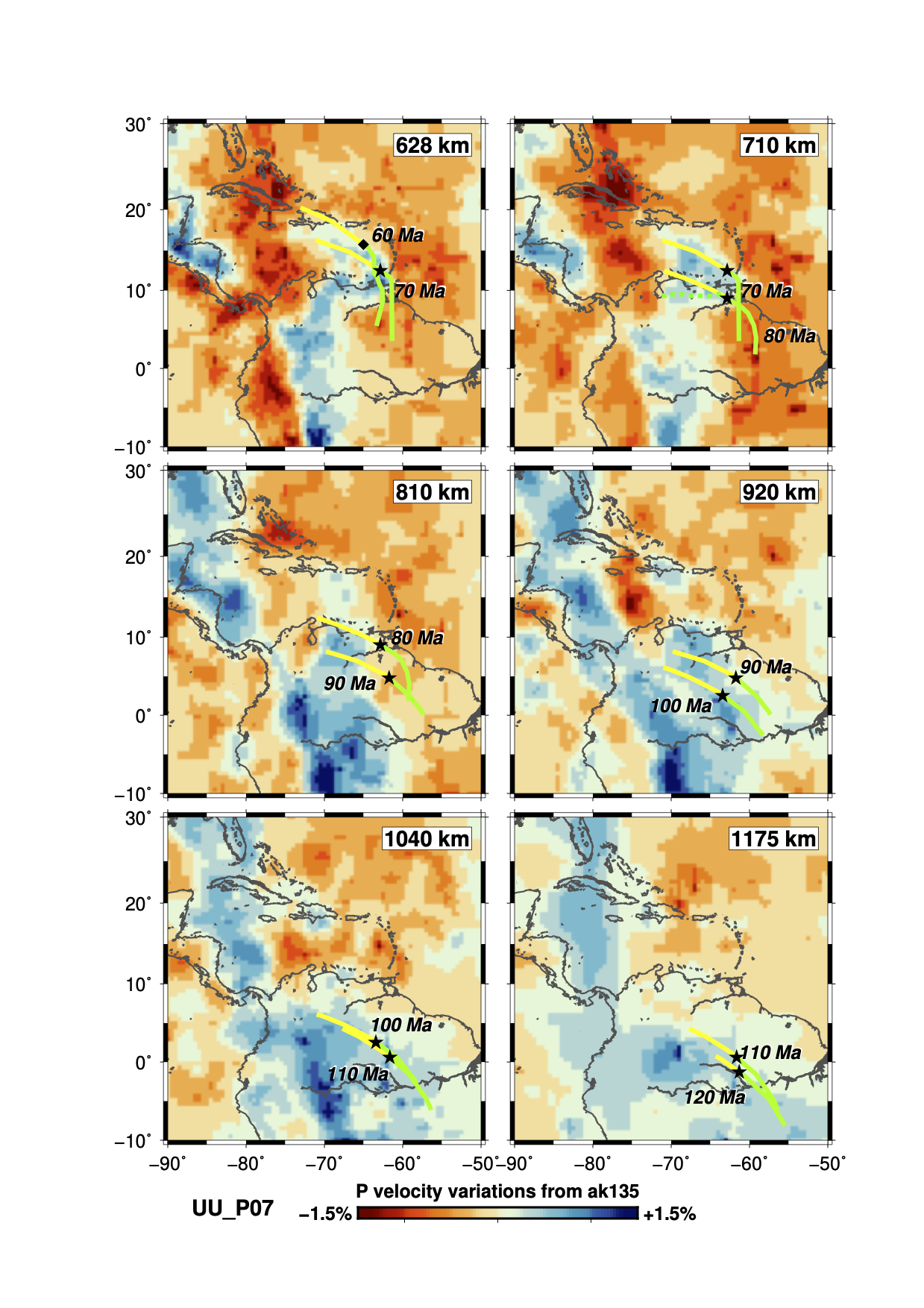
***

**Supplementary Figure** **15**. **Shallow lower mantle structure from UU-P07** ^33,13^ **with our reconstructed trench positions from case1**. Velocity anomalies are relative to global model AK135 ^37^. Coloured lines show our reconstructed slab positions, assuming vertical sinking, at the labelled times: in yellow, slabs subducted along the northern GAC and in green, slabs subducted along the southern GAC, with the black star marking the position of the (at subduction time) actively spreading ridge of the Proto-Caribbean, black diamond marking the ridge when inactive at the time of subduction.


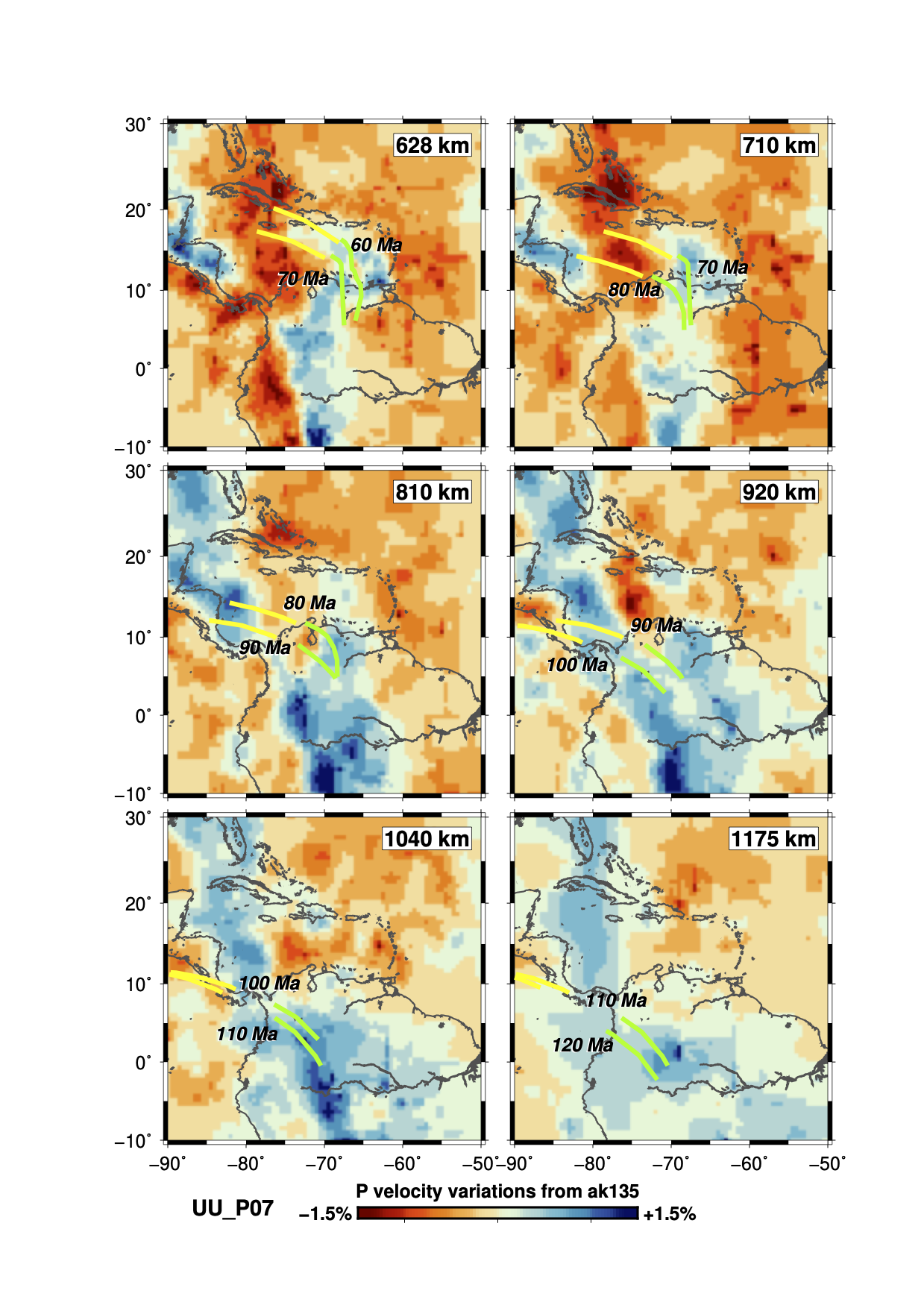


**Supplementary Figure 16**. **Shallow lower mantle structure from UU-P07** ^33,13^ **with our reconstructed trench positions using our hybrid scenario**. In this case 3 scenario, the slabs move with NAM or SAM (depending on whether subducted material originated north or south of the Proto-Caribbean ridge) until 50 Ma and sink vertically after. Velocity anomalies are relative to global model AK135 ^37^. Coloured lines show our reconstructed slab positions at the labelled times: in yellow, slabs subducted along the northern GAC and in green, slabs subducted along the southern GAC, with the black star marking the position of the (at subduction time) actively spreading ridge of the Proto-Caribbean, black diamond marking the ridge when inactive at the time of subduction.
