# Supplementary material for: Subduction history of the Caribbean from upper-mantle seismic imaging and plate reconstruction
Source: Nat Commun. 2021 Jul 9;12:4211. doi: 10.1038/s41467-021-24413-0 (PMC8270990; doi:10.1038/s41467-021-24413-0)
Supplement: Supplementary file 2 — Description of Additional Supplementary Files [file 41467_2021_24413_MOESM2_ESM.pdf]

Description of Additional Supplementary Files

File name: Supplementary Movie 1
Description: Video of plate reconstruction for Caribbean Plate region for the period 120Ma-Present run in G-Plates with annotations for key tectonic events. Reconstruction from Müller et al. [2019] (ref. 18) with modifications discussed in the main text (see also Figure 4 of the main text for detailed discussion of time period captured in this video). In yellow: Cuban part of the GAC. In green: Aves/Leeward Antilles part of the GAC, both of which move in a mantle reference frame. Over-riding Caribbean plate shown in pink from 85 Ma (prior to this only the leading edge of the Caribbean Plate is shown). Flowlines (arrowed lines) replicate major fracture zones within the Central/Equatorial Atlantic and define the orientation of spreading within the protoCaribbean
